# Trends in incidence and survival in patients with gastrointestinal neuroendocrine tumors: A SEER database analysis, 1977-2016

**DOI:** 10.3389/fonc.2023.1079575

**Published:** 2023-01-26

**Authors:** Miao Liu, Lingge Wei, Wei Liu, Shupeng Chen, Meichao Guan, Yingjie Zhang, Ziyu Guo, Ruiqi Liu, Peng Xie

**Affiliations:** ^1^ Department of Nuclear Medicine, The Third Hospital, Hebei Medical University, Shijiazhuang, Hebei, China; ^2^ Department of Nuclear Medicine, Hebei General Hospital, Shijiazhuang, Hebei, China

**Keywords:** gastrointestinal neuroendocrine tumors, incidence, survival, relative, risk factors

## Abstract

**Objectives:**

We aimed to determine trends in incidence and survival in patients with gastrointestinal neuroendocrine tumors (GI-NETs) from 1977 to 2016, and then analyze the potential risk factors including sex, age, race, grade, Socioeconomic status (SES), site, and stage.

**Methods:**

Data were obtained from Surveillance, Epidemiology, and End Results Program (SEER) database. Kaplan-Meier survival analysis, relative survival rates (RSRs), and Cox proportional risk regression model were used to evaluate the relationship between these factors and prognosis.

**Results:**

Compared with other sites, the small intestine and rectum have the highest incidence, and the appendix and rectum had the highest survival rate. The incidence was higher in males than in females, and the survival rate in males was close to females. Blacks had a higher incidence rate than whites, but similar survival rates. Incidence and survival rates were lower for G3&4 than for G1 and G2. Age, stage, and grade are risk factors.

**Conclusions:**

This study described changes in the incidence and survival rates of GI-NETs from 1977 to 2016 and performed risk factor analyses related to GI-NETs.

## 1 Introduction

Neuroendocrine tumors (NETs) are heterogeneous malignancies arising from the diffuse neuroendocrine system. NETs frequently originate in the gastroenteropancreatic (GEP) tract and the bronchopulmonary tree, and the incidence has steadily increased in the last 3 decades (1). Gastroenteropancreatic NETs (GEP-NETs) include gastrointestinal NETs (GI-NETs) and pancreatic NETs (pNETs). GI-NETs currently account for 80% of all primary NETs. Notably, the GI-NETs incidence and prevalence have been increasing in the United States. Recent studies indicated the highest incidence of GI-NETs to be 3.56 per 100,000 population (2).

GI-NETs can occur in the stomach, colon, rectum, appendix, and small intestine. Recent studies have shown that the overall incidence and prognosis of patients with GI-NETs are related to the location and stage of the tumor (3). However, there is seldom a comprehensive analysis of GI-NETs in a large population, so more epidemiological studies are needed to analyze and evaluate the clinical characteristics of GI-NETs, providing important information for rapid diagnosis, accurate treatment, and effective prognosis assessment.

The epidemiological statistical analysis variables for most diseases include age, sex, and race. In addition, pathology grade and Socioeconomic status (SES) are also important. Pathological grade analysis of tumors may be helpful for treatment selection and prognosis assessment. It has been reported that SES is related to timely and effective access to medical resources by patients with malignant tumors. People with high SES can afford more testing and treatment costs. Therefore, to describe overall morbidity and survival trends and to assess factors associated with the survival and prognosis of GI-NETs, we analyzed 7 variables, including age, sex, race, SES, pathological grade, site, and stage, in a large population in the United States.

## 2 Material and methods

### 2.1 Data selection

All data on GI-NETs patients from 9 original Surveillance, Epidemiology, and End Results Program (SEER) over 4 decades (1977–2016) were collected from the SEER∗ Stat software program (version 8.4.0). The original 9 SEER sites include the states of San Francisco-Oakland (SF-O) Standard Metropolitan Statistical Area (SMSA), Connecticut, Hawaii, Iowa, New Mexico, Utah, Atlanta (metropolitan), Detroit (metropolitan), and Seattle (Puget Sound). The database, which registers about 400,000 cancer cases and stores cancer data for one-third of the U.S. population, is a great aid to medical researchers in the statistical analysis of diseases. Oncology and histologic codes of GI-NETs were determined by the International Classification of Diseases for Oncology (3rd editions) (ICD-O-3) codes. Primary locations of tumors of the gastrointestinal tract: C16.0-C20.9. Therefore, GI-NETs mainly include the following diseases: gastrinoma, malignant (8153/3); somatostatinoma, malignant (8156/3); carcinoid tumor, NOS (8240/3); enterochromaffin cell carcinoid (8241/3); enterochromaffin-like cell tumor, malignant (8242/3); goblet cell carcinoid (8243/3); mixed adenoneuroendocrine carcinoma (8244/3); adenocarcinoma tumor (8245/3); neuroendocrine carcinoma, NOS (8246/3); and atypical carcinoid tumor (8249/3). Data analyzed in this study included the incidence and relative survival rates (RSRs) of GI-NETs. Patients diagnosed with GI-NETs between 1977 and 2016 were enrolled and continued active follow-up was maintained. And excluded the patients diagnosed by autopsy or as stated on a death certificate. The time of follow-up for all analyses was from the date of diagnosis until death, the date of the last contact, or the end of the study period.

### 2.2 Variable definition

Sex, age, race, grade, SES, site, and stage were the patient variables examined in this study. The socioeconomic status (SES) of the area was determined using the county poverty rate (4, 5), which is the percentage of persons in the county living below the national poverty threshold in the Census 2000 (The 0-9.99%, 10%-19.9%, and 20%-56.92% of persons whose incomes are below the poverty 2000 level are defined as low-poverty, medium-poverty, and high-poverty, these can be selected in the SEER*Stat software) (6). The patients in the current study were classified by socioeconomic status (SES) (low-poverty, medium-poverty, high-poverty), sex, race (White, Black, and others), and age at diagnosis (0-44, 45-59, 60-74, and 75^+^y). We used SEER histologic grade information to classify cases as grade (G) 1, well-differentiated; G2, moderately differentiated; G3, poorly differentiated; and G4, undifferentiated or anaplastic (7). Because of the small number of patients with low differentiation, we combined G3 and G4 into 1 category for all analyses. The stage of the tumor uses the “Combined Summary Stage (2004+) new” based on SEER, including localized, regional, and distant. Localized disease is defined as NETs that have not spread outside the wall of the primary organ, regional metastasis includes NETs that have spread beyond the wall into surrounding tissue or lymph nodes, and distant metastasis includes NETs that have spread to tissue or organs away from the primary organ (3).

### 2.3 Statistical analysis

We categorized all data of incidences and relative survival rates (RSRs) on GI-NETs patients by period: 1977–1986, 1987–1996, 1997–2006, and 2007-2016. The 12-month, 60-month, and 120-month RSRs were demonstrated by survival rate curves. The two-tailed log-rank test was used to access the difference in survival, using the Kaplan–Meier curves generated by GraphPad Prism 5.0. A two-tailed *p*-value < 0.05 was considered statistically significant. The Cox proportional hazard univariate and multivariate models were used to identify survival risk factors, including sex, age, race, grade, SES, site, and stage for the entire cohort.

## 3 Results

### 3.1 Trends in GI-NETs incidence at the nine original SEER sites over four decades from 1977-2016

**Figure 1:**
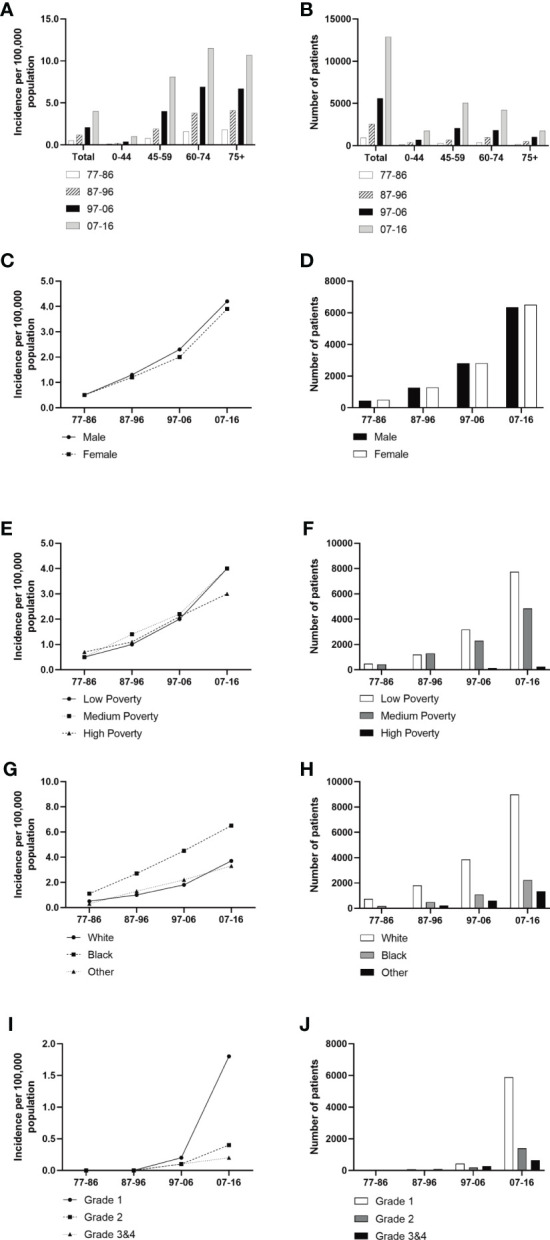
Incidence of Patients diagnosed with GI-NETs at the original nine SEER sites between 1977 and 2016. The incidence and number of GI-NETs cases are shown by age group (total and age 0-44 years, 45-59 years, 60-74 years, and over 75 years) and four-time periods. Incidence **(A, C, E, G, I)** and number **(B, D, F, H, J)** of GI-Nets cases were grouped by sex, SES, race, and grade, respectively.

A total of 21,983 patients diagnosed with GI-NETs between 1977 and 2016 in the SEER program of the National Cancer Institute at the nine original registry sites were collected. As indicated in **Figure 1** and **Supplementary Table 1**, the GI-NETs incidence in the four decades continually increased (0.5 per 100,000 from 1977 to 1986, 1.2 per 100,000 from 1987 to 1996, 2.1 per 100,000 from 1997 to 2006, and 4.0 per 100,000 from 2007 to 2016). Similar trends were observed across all age groups in the study over the past 40 years, with the highest incidence in the 75+ age group in the first two decades and the highest incidence in the 60-74 age group in the last two decades.

### 3.2 GI-NETs incidence by sex, race, SES, grade, and site

**Figure 2:**
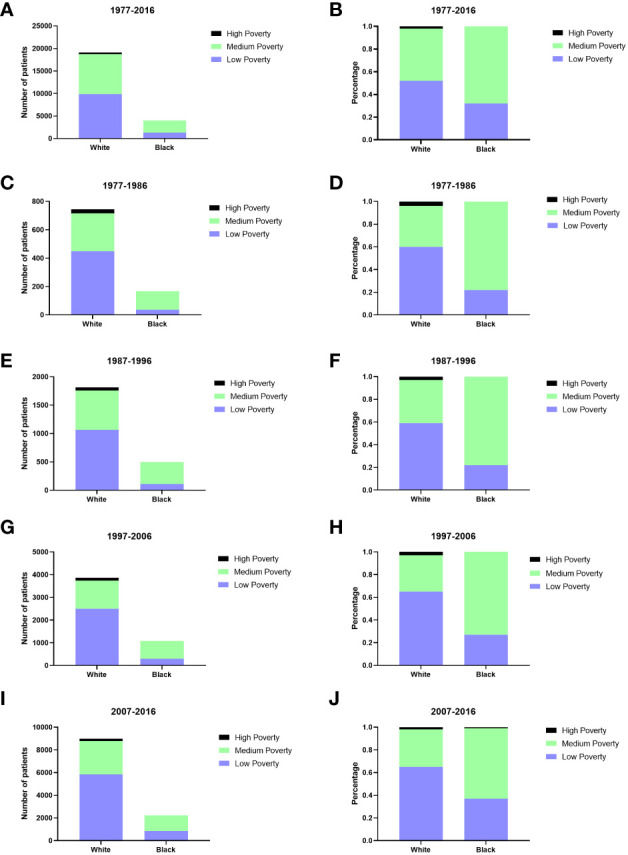
The numbers of patients with GI-NETs of SES in different races across four decades **(A, C, E, G, I)**; Changes in the distribution of SES in different races across four decades **(B, D, F, H, J)**.

Males had a higher incidence of GI-NETs per 100,000 people than females (**Figure 1**). In race groups, the incidence of Blacks was higher than Whites and other races, and from 1977 to 2006, the rate of Blacks was approximately 2-fold higher than the average Whites (**Supplementary Table 1**). But there were significant racial differences, with whites in particular far outnumbering blacks. The medium-poverty group showed a slightly higher GI-NETs incidence than that of the low- and high-poverty groups. GI-NETs incidence per 100,000 in all poverty groups exhibited an increasing trend (from 0.5 to 1.0 to 2.0 to 4.0 in the low-poverty group, from 0.5 to 1.4 to 2.2 to 4.0 in the medium-poverty group and from 0.7 to 1.1 to 2.1 to 3.0 in the high-poverty group). In addition, we also analyzed the distribution characteristics of SES in different ethnic groups. The share of rich and poor by race has remained nearly constant in each decade (**Figure 2**). The incidence of the G1 group increased significantly in the last decade and the number of patients increased dramatically.

**Figure 3:**
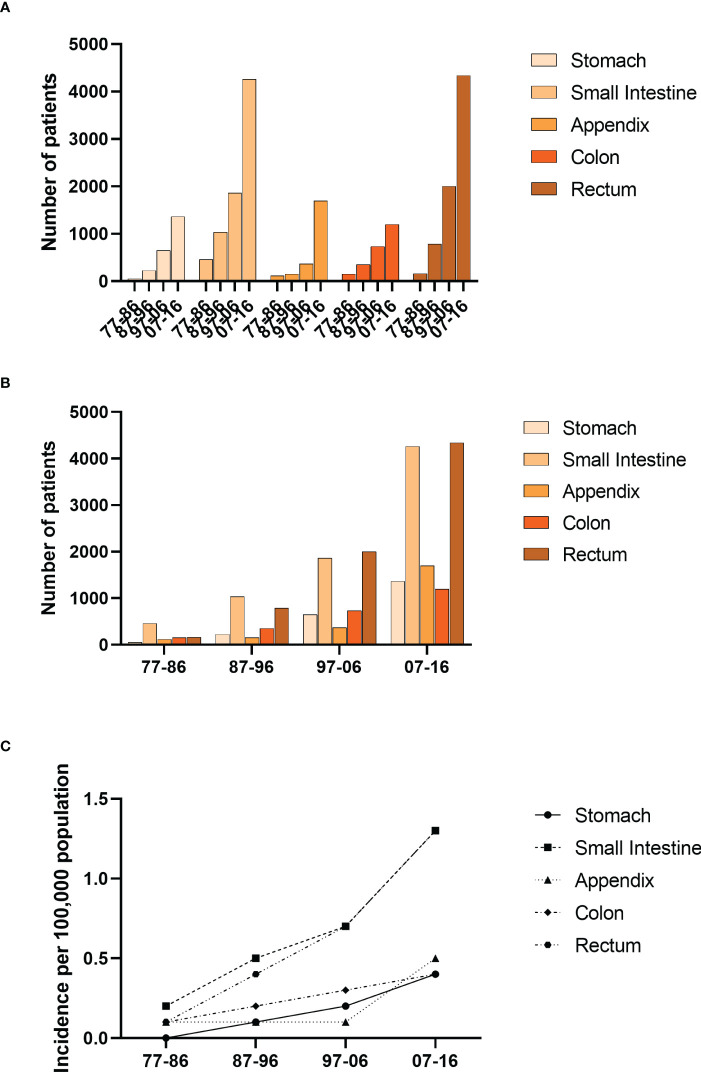
Incidence of Patients diagnosed with GI-NETs at the original nine SEER sites between 1977 and 2016. The number **(A, B)** and incidence **(C)** of GI-Nets cases are shown by site group (stomach, small intestine, appendix, colon, rectum) and four-time periods.

We divided the pathogenic sites of GI-NETs into five parts, including the stomach, small intestine, appendix, colon, and rectum. The incidence of GI-NETs in each site has increased significantly over the past four decades. The small intestine and rectum have the highest incidence in each decade (**Figure 3**). The incidence was highest in the last decade compared to the previous three (from 0.2 to 0.5 to 0.7 to 1.3 in the small intestine and from 0.1 to 0.4 to 0.7 to 1.3 in the rectum) (**Supplementary Table 1**).

### 3.3 Relative survival estimates for the 9 SEER sites over four decades in 1977-2016

**Figure 4:**
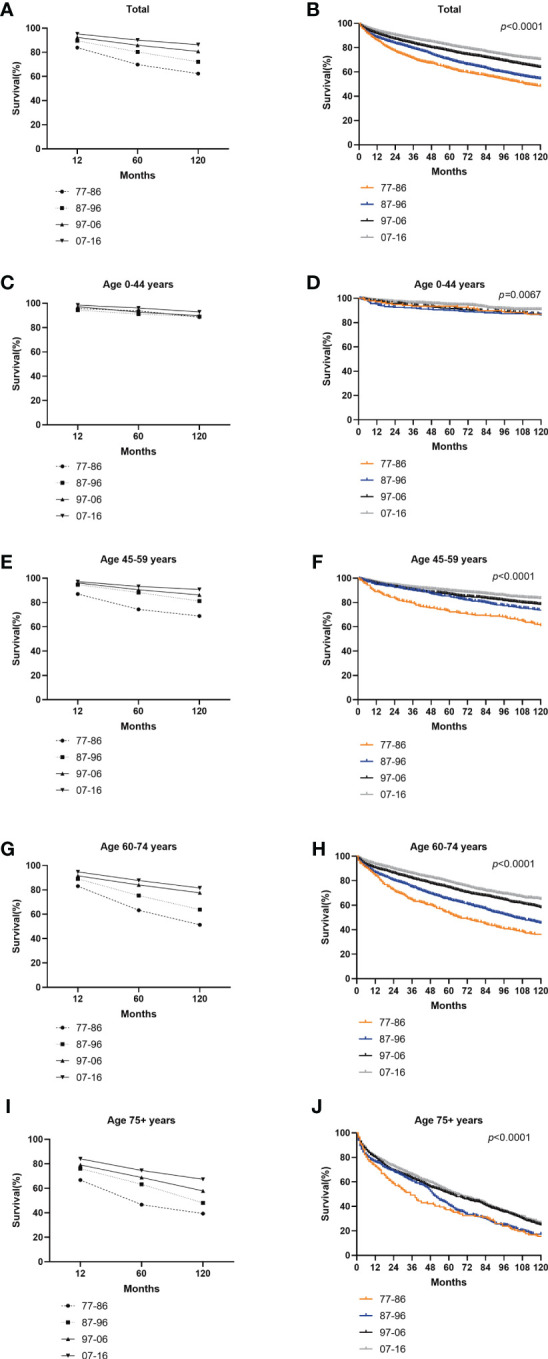
Trends in 10-year relative survival **(A, C, E, G, I)** and Kaplan-Meier survival analysis **(B, D, F, H, J)** for PATIENTS with GI-NETs during 1977-1986 (orange), 1987-1996 (blue), 1997-2006 (black), and 2007-2016 (gray), grouped by age (total and age 0-44 years, 45-59 years, 60-74 years and over 75 years).

The RSRs and survival times of patients with GI-NETs across the four decades improved for each age group analyzed (**Figure 4**). The one-year RSR gradually increased over time (83.9% from 1977 to 1986, 89.5% from 1987 to 1996, 92.4% from 1997 to 2006, and 95.3% from 2007 to 2016; P < 0.0001 for each decade) (**Table 1**). Kaplan-Meier survival analysis indicated increases in survival time over the four decades for all age groups. The 5-year RSR increased from 69.9% to 80.3% to 85.9% to 90.1% over the four decades. The 10-year RSR increased from 62.4% to 72.1% to 80.7% to 86.3% over the fourth decade. The data indicate that the gap between five-year RSRs and 10-year RSRs has increased over the past four decades in the 45-59 and 60-74 age groups. (**Figure 4** and **Table 1**).

**Table 1:** Relative survival rates of GI-NETs during the periods of 1977-1986, 1987-1996, 1997-2006, and 2007-2016 at nine SEER sites.

Age group	Decade			
	1977-1986	1987-1996	1997-2006	2007-2016
12-Mo Rs				
All	83.9 ± 1.4 (801)	89.5 ± 0.7 (2105)***	92.4 ± 0.4 (4623)**	95.3 ± 0.2 (10422)***
0-44	95.7 ± 1.8 (133)	94.4 ± 1.2 (363)	97.2 ± 0.7 (675)	98.5 ± 0.3 (1665)
45-59	87.0 ± 2.2 (255)	94.7 ± 1.0 (624)**	96.1 ± 0.5 (1846)	97.2 ± 0.3 (4465)
60-74	83.0 ± 2.4 (288)	89.0 ± 1.3 (752)*	91.5 ± 0.8 (1425)	94.8 ± 0.5 (3156)**
75+	66.8 ± 4.7 (125)	76.1 ± 2.5 (366)*	79.1 ± 1.8 (677)	84.1 ± 1.3 (1136)**
60-Mo Rs				
All	69.9 ± 1.9 (801)	80.3 ± 1.1 (2105)***	85.9 ± 0.7 (4623)***	90.1 ± 0.4 (10422)***
0-44	93.8 ± 2.2 (133)	91.1 ± 1.6 (363)	92.7 ± 1.1 (675)	96.1 ± 0.6 (1665)
45-59	74.4 ± 3.0 (255)	88.3 ± 1.5 (624)**	90.5 ± 0.8 (1846)	93.3 ± 0.5 (4465)
60-74	63.3 ± 3.4 (288)	75.4 ± 2.0 (752)**	84.0 ± 1.3 (1425)**	87.7 ± 0.8 (3156)
75+	46.7 ± 6.4 (125)	63.3 ± 4.0 (366)*	69.8 ± 2.8 (677)	74.6 ± 2.3 (1136)
120-Mo Rs				
All	62.4 ± 1.8 (801)	72.1 ± 1.4 (2105)*	80.7 ± 0.8 (4623)***	86.3 ± 0.7 (10422)***
0-44	88.6 ± 3.1 (133)	89.0 ± 1.9 (363)	89.8 ± 1.3 (675)	92.9 ± 1.1 (1665)
45-59	68.9 ± 3.4 (255)	81.2 ± 1.9 (624)	86.3 ± 1.0 (1846)	90.8 ± 0.8 (4465)
60-74	51.3 ± 4.0 (288)	63.8 ± 2.5 (752)	77.6 ± 1.7 (1425)***	81.5 ± 1.4 (3156)
75+	39.4 ± 8.4 (125)	48.1 ± 5.4 (366)	57.9 ± 3.9 (677)	67.4 ± 3.9 (1136)

Data are represented as mean ± standard error of the mean, with the number of patients in parentheses. Mo, month; RS, relative survival; SEM, standard error of the mean.

*P < 0.01 for comparisons with the preceding decade.

**P < 0.001 for comparisons with the preceding decade.

***P < 0.0001 for comparisons with the preceding decade.

**Figure 5:**
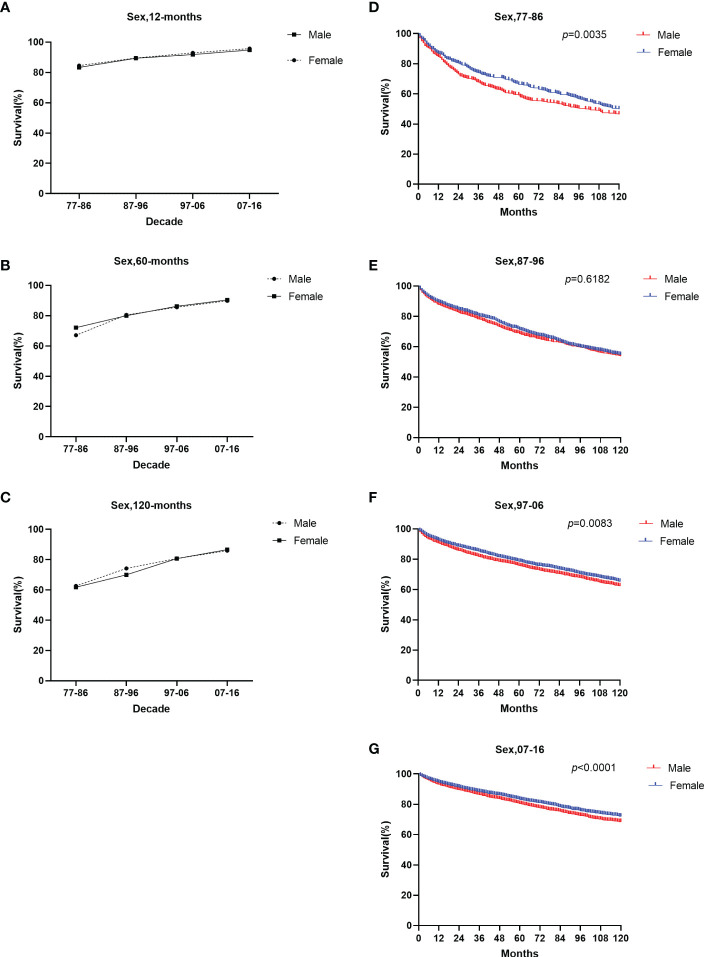
Trends in relative survival rate **(A–C)** and Kaplan–Meier survival curves **(D–G)** for patients with GI-NETs at 9 SEER sites according to sex group (male and female) in 1977–1986, 1987–1996, 1997–2006, and 2007-2016.

The survival rate in both sexes over the four decades improved (**Figure 5**). Females showed a slightly higher 12-month RSR than males from 1977 to 2016 (84.5% for females vs. 83.3% for males from 1977 to 1986, 89.5% for females vs. 89.4% for males from 1987 to 1996, 92.9% for females vs. 91.8% for males from 1997 to 2006, 95.7% for females vs. 94.8% for males from 2007 to 2016) (**Supplementary Table 2**). However, from 1987 to 1996, the 60-month RSR of males was slightly higher than that of females (80.5% vs. 80.0%). The 120-month RSR of males was slightly higher than that of females in the first three decades (61.8% for females vs. 62.6% for males from 1977 to 1986, 70.0% for females vs. 74.2% for males from 1987 to 1996, 80.7% for females vs. 80.8% for males from 1997 to 2006). Only in the fourth decade females have a higher 120-month RSR than males (86.6% for females vs. 85.9% for males from 2007 to 2016). The results showed that gender was statistically significant in the first decade, the third decade, and the last decade (p = 0.0035 in 1977–1986, p=0.0083 in 1997–2006, p <0.0001 in 2007-2016) (**Figure 5**). Notably, we found no significant sex disparities in age groups at 12- and 60- months of RSR. Therefore, the improvement in the overall survival rate of patients of different genders may be due to the improvement in social medical conditions and people’s concerns.

### 3.4 Survival of GI-NETs in different race, SES, grade, and site groups

**Figure 6:**
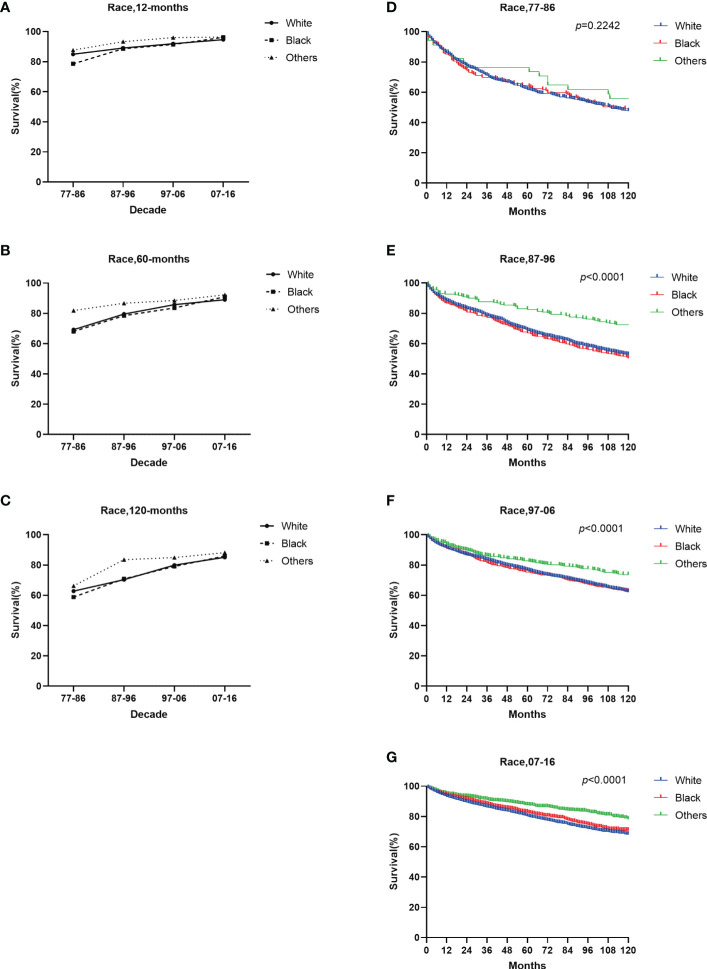
Trends in relative survival rate **(A–C)** and Kaplan–Meier survival curves **(D–G)** for patients with GI-NETs at 9 SEER sites according to race group (whites, blacks, and others) in 1977–1986, 1987–1996, 1997–2006, and 2007-2016.

White patients exhibited a slightly higher 12-month RSR than Black patients in the first three decades (84.9% vs. 78.6% from 1977 to 1986, 89.1% vs. 88.6% from 1987 to 1996, 91.9% vs. 91.4% from 1997 to 2006) but the last decade was the opposite (94.7% vs. 96.2% from 2007 to 2016) (**Supplementary Table 3**). A similar tendency over time was observed in the 60-month survival rates. Overall, whites have slightly higher survival rates than blacks. The 12-, 60-, and 120-month RSR of other race groups was significantly higher than Whites and Blacks over the four decades. This is due to the low number of other ethnic groups (**Figure 6** and **Supplementary Table 3**).

All SES groups showed improvement in survival rate across the four decades (**Supplementary Figure 1**). The low-poverty group consistently exhibited the highest 12-, 60-, and 120-month RSRs, except the 12-month RSR group in the second decade. In comparison with the low poverty group, the medium poverty groups of the 60-month RSR in the penultimate decade and 120-month RSR in the fourth decade were statistically significant (91.8%vs.83.8%%, p<0.0001;87.6%vs.83.9%, p<0.001) (**Supplementary Table 4**). Notably, Different SES groups were distributed differently among blacks and whites. There were more whites than blacks in the low poverty group (52% vs.32%), and more blacks than whites in the middle poverty group (68% vs. 46%) (**Figure 2**). The difference in survival between whites and blacks reflects the difference between the different SES groups, which have a certain connection. A similar trend was indicated in the Kaplan-Meier survival analysis for the three SES groups over the four decades. Lower poverty may be associated with higher survival.

Differences in long-term survival in pathologic grades have increased over the past 40 years (p=0.0005 in 1977–1986, p < 0.0001 in 1987–1996, p < 0.0001 in 1997–20066, and p < 0.0001 in 2007–2016) (**Supplementary Figure 2**). In grade groups, the G3&4 group consistently exhibited the lowest 12-, 60-, and 120-month RSRs, whereas the G1 group consistently showed the highest survival rates, except for the 12-month RSR group in the first decade. Overall, the RSR gap between G1 and G2 groups gradually narrowed, while the RSR gap between G3&4 groups continued to be significantly lower than that between the G1 and G2 groups. Kaplan Meier survival curve and log-rank test showed that the survival rate of low-grade GI-NETs increased year by year, suggesting that low-grade GI-NETs treatment was satisfactory. Although the incidence of G3&4 was low, there was little improvement in 40-year long-term survival (**Supplementary Table 5**). We can’t ignore poorly differentiated GI-NETs. Therefore, clinical and medical workers need to pay more attention to this disease, to achieve a complete grasp of the disease.

The 12-, 60-, and 120-month RSR of the colon group was significantly lower than the remaining four groups over the four decades (**Supplementary Figure 3** and **Supplementary Table 6**). The same trend was observed in all age groups. And the 12-, 60-, and 120-month RSR of the appendix group during the first decade was the highest. However, during the next three decades, the 12-, 60-, and 120-month RSR in the rectum was highest and remained stable. There was almost no significant difference in RSR between the stomach and the other four sites during the first decade. In the 75+ age group of the second decade, the 12-month RSR of the small intestine and rectum was significantly higher than that of the stomach (52.3% vs. 79.4, p<0.01;52.3% vs. 90.3%, p<0.001). Over the next three decades, rectum relative survival rates at 12-, 60-, and 120- months increased significantly. Differences in long-term survival have gradually diminished over the past four decades (**Supplementary Figure 4**).

Cox risk-proportional regression model assessed the prognostic value of seven risk factors (sex, age, race, SES, grade, stage, and site) for GI-NETs. Due to the incomplete update of the database, we have analyzed the effect of tumor stage on prognosis only in the last two decades. Analysis showed that stage, age, and pathological grade are risk factors for the prognosis of patients with GI-NETs. Data analysis results showed that the hazard ratio of the stage (p<0.001 and p<0.001), age (p=0.015, p < 0.001, p < 0.001 and p < 0.001 in 1977–2016), and grade (p=0.046, p < 0.001, p < 0.001, and p < 0.001 in 1977–2016) were greater than 1, indicating that the higher the stage, the shorter the survival time. Similarly, the older the age, the shorter the survival time; the less differentiated, the shorter the survival time. Other risk factors, such as race, were a risk factor for GI-NETs over the first, second, and last decades and were no longer a risk factor for GI-NETs in the third decade (p=0.012, HR=3.081, 95% CI 1.280–7.418 in 1977-1986, p=0.008, HR=2.365, 95% CI 1.252–4.470 in 1987-1996, p<0.001, HR=1.349, 95% CI 1.166-1.562 in 2007-2016). In addition, sex was not a risk factor for GI-NETs from 1977 to 1996, but became a risk factor for GI-NETs in the following two decades, influencing patient outcomes (p=0.011, HR=1.396, 95% CI 1.081-1.804 in 1997-2006, p<0.001, HR=1.220, 95% CI 1.108-1.344 in 2007-2016). Site of GI-NETs in the last two decades as a risk factor affecting GI-NETs prognosis. (**Table 2**).

**Table 2:** Summary data for Cox regression analysis of survival in patients with GI-NETs from 1977 to 2016 at nine SEER sites.

Variable	Relative risk (95% CI)	*P* value
All 1977-1986		
Univariate		
Sex		
Female	1	
Male	1.223 (0.652-2.295)	0.530
Age	1.043 (1.019-1.068)	<0.001
Race		
White	1	
Black	3.786 (1.650-8.685)	0.002
Other	0.541 (0.129-2.277)	0.402
SES		
Low poverty	1	
Medium poverty	1.071 (0.566-2.027)	0.832
High poverty	2.690 (0.342-21.158)	0.347
Grade		
G1	1	
G2	2.477 (0.863-7.113)	0.092
G3&4	3.071 (1.502-6.277)	0.002
Site		
Stomach	1	
Small intestine	0.562 (0.169-1.872)	0.348
Appendix	0.294 (0.072-1.204)	0.089
Colon	0.703 (0.233-2.120)	0.532
Rectum	0.339 (0.093-1.237)	0.101
Multivariate		
Age	1.033 (1.006-1.060)	0.015
Race		
White	1	
Black	3.081 (1.280-7.418)	0.012
Other	0.926 (0.208-4.117)	0.920
Grade		
G1	1	
G2	2.124 (0.704-6.402)	0.181
G3&4	2.170 (1.015-4.642)	0.046
All 1987-1996		
Univariate		
Sex		
Female	1	
Male	0.912 (0.681-1.221)	0.537
Age	1.051 (1.038-1.065)	<0.001
Race		
White	1	
Black	1.142 (0.761-1.714)	0.521
Other	1.858(1.004-3.439)	0.049
SES		
Low poverty	1	
Medium poverty	1.090 (0.814-1.460)	0.564
High poverty	0.000	0.953
Grade		
G1	1	
G2	1.339 (0.894-2.005)	0.156
G3&4	3.066 (2.167-4.338)	<0.001
Site		
Stomach	1	
Small intestine	0.455 (0.279-0.740)	0.002
Appendix	0.248 (0.101-0.613)	0.003
Colon	0.735 (0.457-1.181)	0.203
Rectum	0.497 (0.285-0.867)	0.014
Multivariate		
Age	1.048 (1.035-1.063)	<0.001
Race		
White	1	
Black	1.285 (0.838-1.969)	0.250
Other	2.365 (1.252-4.470)	0.008
Grade		
G1	1	
G2	1.095 (0.715-1.678)	0.676
G3&4	2.258 (1.555-3.278)	<0.001
Site		
Stomach	1	
Small intestine	0.662 (0.400-1.095)	0.108
Appendix	0.499 (0.198-1.254)	0.139
Colon	0.777 (0.475-1.270)	0.314
Rectum	0.740 (0.421-1.304)	0.298
All 1997-2006		
Univariate		
Sex		
Female	1	
Male	1.261 (1.069-1.486)	0.006
Age	1.051 (1.044-1.059)	<0.001
Race		
White	1	
Black	1.184 (0.941-1.488)	0.149
Other	0.904 0.665-1.228)	0.517
SES		
Low poverty	1	
Medium poverty	1.097 (0.927-1.298)	0.283
High poverty	0.731 (0.428-1.248)	0.251
Grade		
G1	1	
G2	1.922 (1.543-2.394)	<0.001
G3&4	4.750 (3.917-5.760)	<0.001
Site		
Stomach	1	
Small intestine	0.682 (0.539-0.863)	0.001
Appendix	0.613 (0.399-0.941)	0.025
Colon	1.234 (0.971-1.567)	0.085
Rectum	0.369 (0.269-0.506)	<0.001
Stage		
Localized	1	
Regional	1.678 (1.232-2.285)	0.001
Distant	4.508 (3.318-6.125)	<0.001
Multivariate		
Sex		
Female	1	
Male	1.396 (1.081-1.804)	0.011
Age	1.045 (1.034-1.057)	<0.001
Grade		
G1	1	
G2	1.745 (1.241-2.452)	0.001
G3&4	3.278 (2.369-4.536)	<0.001
Site		
Stomach	1	
Small intestine	0.516 (0.345-0.771)	0.001
Appendix	0.458 (0.233-0.902)	0.024
Colon	0.619 (0.413-0.928)	0.020
Rectum	0.541 (0.339-0.861)	0.010
Stage		
Localized	1	
Regional	1.194 (0.842-1.693)	0.320
Distant	4.253 (2.952-6.126)	<0.001
All 2007-2016		
Univariate		
Sex		
Female	1	
Male	1.168 (1.062-1.285)	0.001
Age	1.066 (1.062-1.070)	<0.001
Race		
White	1	
Black	0.820 (0.711-0.946)	0.007
Other	0.719 (0.596-0.866)	0.001
SES		
Low poverty	1	
Medium poverty	1.017 (0.920-1.125)	0.736
High poverty	1.077 (0.749-1.548)	0.689
Grade		
G1	1	
G2	1.547 (1.358-1.762)	<0.001
G3&4	8.468 (7.587-9.450)	<0.001
Site		
Stomach	1	
Small intestine	.826 (0.708-0.964)	0.015
Appendix	0.456 (0.368-0.564)	<0.001
Colon	1.728 (1.460-2.046)	<0.001
Rectum	0.377 (0.312-0.457)	<0.001
Stage		
Localized	1	
Regional	2.040 (1.804-2.306)	<0.001
Distant	6.260 (5.580-7.024)	<0.001
Multivariate		
Sex		
Female	1	
Male	1.220(1.108-1.344)	<0.001
Age	1.058(1.054-1.063)	<0.001
Race		
White	1	
Black	1.349 (1.166-1.562)	<0.001
Other	0.958 (0.792-1.159)	0.658
Grade		
G1	1	
G2	1.297 (1.137-1.479)	<0.001
G3&4	4.443 (3.889-5.076)	<0.001
Site		
Stomach	1	
Small intestine	0.636 (0.536-0.754)	<0.001
Appendix	0.654 (0.525-0.814)	<0.001
Colon	0.934 (0.783-1.114)	0.446
Rectum	0.647 (0.532-0.786)	<0.001
Stage		
Localized	1	
Regional	1.402 (1.220-1.611)	<0.001
Distant	4.245 (3.712-4.856)	<0.001

95% CI, 95% confidence interval; SES, socioeconomic status.

## 4 Discussion

The GI-NETs incidence and the RSRs (relative survival rates) for GI-NETs both increased in each decade from 1977 to 2016. In particular, the number of GI-NETs had increased significantly over the past decade (**Figure 1**). Across all the variables we looked at, the gap in long-term survival narrowed. However, ten-year relative survival remained very low for the occurrence of GI-NETs in the colon, poorly differentiated and undifferentiated GI-NETs. Relative survival rates have ranged from 13.7% to 27.1% over the past four decades, indicating an urgent need to develop effective therapies to improve this situation to significantly improve survival in patients with poorly differentiated GI-NETs.

In our population-based study, the incidence of GI-NETs has increased dramatically over the past four decades. From the first decade to the fourth decade, the incidence increased eightfold from 0.5 to 4.0. This may be related to the fact that there was little understanding of GI-NETs in the past, and in 2000 WHO classification published, carcinoid was used separately from neuroendocrine neoplasms and neuroendocrine neoplasms for the first time, which made the classification of endocrine neoplasms clearer (8). The most significant change in 2019 WHO classification of digestive tumors is the neuroendocrine tumor classification system (9). In addition, the increased incidence may be due to the increased prevalence and use of gastrointestinal endoscopy, resulting in a higher detection rate of GI-NETs (10). With the development of medical technology, in addition to conventional imaging examinations such as CT and MRI, more and more imaging techniques such as SSTR positron emission tomography/computed tomography (PET/CT) using 68Ga-labeled somatostatin analog (11–13) and endoscopic ultrasonography (14, 15), have been used to detect tumors. These tests have greatly increased the detection of GI-NETs. With the improvement in people’s living standards, people pay more attention to their health status, which makes them sensitive to the possible early symptoms of GI-NETs. The widespread and vigorous promotion of physical examination has also made it important to detect tumors earlier, especially in the early and asymptomatic stages of the disease.

The overall incidence of GI-NETs per 100,000 people increased significantly from 0.5 to 1.2 to 2.1 to 4.0 per decade. And patients over 60 years old account for the majority of the population. At the same time, the incidence of GI-NETs was higher in men than in women per 100,000 people in the study, which may be because men smoke more than women. Based on one population study, smoking may increase the risk of developing GI-NETs (16). Blacks were more likely to develop GI-NETs than whites and other ethnic groups, and the gap in their incidence widened each year over the 40 years studied. The incidence continued to increase throughout the study period in all SES groups. Compared with the previous three decades, the fourth decade saw the largest increase in all SES groups, especially the low and middle poverty groups. This may be because the low and middle poverty groups pay more and more attention to their health over time, and the detection rate of GI-NETs is higher and higher. However, due to the heavy medical economic burden of the high poverty group, compared with the low and middle poverty groups, it showed steady and continuous growth. With the classification of digestive neuroendocrine tumors by WHO, the incidence of G1 increased significantly compared with poorly differentiated GI-NETs. The G1 has seen the biggest growth over the past decade. This may be due to the clear classification of GI-NETs and the deepening understanding of GI-NETs. Our study showed that the incidence was significantly higher in the small intestine and rectum than in other sites. The results of this study are consistent with those of other studies (17, 18).

Long-term survival has shown a similar trend to the incidence of GI-NETs over the past 40 years (**Figure 1** and **Figure 2**). It is worth noting that the RSR of the 120 months 2007-2016 was 1.38 times that of 1977-1986. Similar to the incidence rate, RSR increases gradually with each decade. Among them, the RSR of 12, 60, and 120 months from 1977 to 1986 showed the most significant increase compared with the RSR of 1987 to 1996 (**Table 1**). This may indicate that since 1987, more attention has been paid to gastrointestinal neuroendocrine tumors, as well as the search for sensitive detection methods and effective treatment. During the last 30 years, the RSR grew steadily each decade. It shows that clinicians are increasingly improving detection rates with more sensitive tests and improving survival rates with more effective treatments. With the continuous improvement of medical treatment, the emergence of new biomarkers and accurate histological assessment and pathological biopsy have greatly improved the survival rate of GI-NETs.

In our study, the prognosis was best in the rectum and appendix. The 60-months survival rates of the rectum and appendix were 97.6% and 90.5%. In addition, the 60-months survival rates of GI-NETs in the other three sites were stomach (83.3%), small intestine (88.6%), and colon (69.9%), respectively. At the same time, our study found that the prognosis of the colon and stomach was worse compared to the rectum and appendix. Long-term survival of the colon and stomach has improved significantly over time but remains low. Moreover, the long-term survival of the rectum and appendix was more stable than that of other sites in our study. With the increased use of colonoscopy and the maturation of treatment modalities, the survival of colonic NET and gastric NET has improved, but it remains in a precarious state. Newer techniques and treatments are needed to further improve survival.

Improvements in long-term survival were observed for both sexes, with females generally having higher survival rates than males (**Figure 5**). The incidence rate for blacks has been significantly higher than for whites and other races over the past four decades, but the survival rate for blacks has been lower than for whites and other races over the last 30 years. Only in the last decade, slightly higher than whites (**Figure 6**). Therefore, the etiology and treatment of black disease need further attention and research. We looked at the socioeconomic status of diagnosed GI-NETs patients over the last 40 years, and survival was higher in the low poverty group (**Supplementary Figure 1**). The higher survival rates of whites compared to blacks may be attributed to the fact that most whites may have sufficient economic conditions to ensure a comfortable living environment and diet, as well as better access to medical services and more accurate diagnosis of diseases than other races. In terms of grade, the incidence of highly differentiated tumors was higher than that of undifferentiated tumors. Survival rates are on a similar trend (**Supplementary Figure 2**). The increasing incidence of poorly differentiated and undifferentiated tumors over the past four decades, while survival remains low, suggests that medical researchers need to pay more attention to the treatment of poorly differentiated and undifferentiated tumors.

Age, stage, and pathological grade were the risk factors for GI-NETs by Cox proportional risk regression model (**Table 2**). Through age grouping comparison, the incidence rate of elderly patients over 60 years old increased significantly, while the survival rate decreased significantly, which may be attributed to the deterioration of physical function, decreased immunity, and poor tolerance to drugs, surgery, and other treatments in elderly patients. At the same time, the elderly suffer from more basic diseases, such as high blood pressure and diabetes, which put a heavy burden on their bodies. Recent studies have shown that more than 80% of GI-NETs patients have metastases by the time they are diagnosed (19). The liver is the most common site of metastasis. For patients with advanced metastasis, there is currently no clinically effective treatment, resulting in a reduced survival rate for these patients (20). Current treatment methods mainly include drug therapy to relieve hormone-related symptoms or syndromes (21, 22) tumor growth control (23, 24) endoscopic therapy (lesions confined to the mucosa and submucosa) (25, 26) gastrointestinal surgery, interventional therapy (mainly for liver metastases) (27, 28) and radionuclide therapy (29, 30). However, these treatments can be too taxing for elderly patients. Although some progress has been made in the treatment of GI-NETs, there is still no relatively safe and effective treatment, especially in elderly patients with metastasis.

Tumor grade was an important prognostic factor by multivariate Cox regression analysis (**Table 2**). The worse the differentiation, the worse the prognosis and the lower the patient’s survival rate. With advances in medical technology, the incidence of G1 GI-NETs has increased steeply in the last decade, probably due to the greater understanding of the nomenclature, classification, and histological and pathological features of GI-NETs (1, 31). In our study, the relative risk of tumor grade was the highest. Patients with highly differentiated GI-NETs can survive for a long time even with metastasis. However, poorly differentiated or undifferentiated GI-NETs are considered to be likely to transition to cancer, leading to a significant reduction in patient survival. Therefore, it is necessary to clarify the tumor grade of patients and carry out close observation and follow-up of patients.

Yao et al. reported an increase in the incidence of neuroendocrine tumors, but there was no significant gender difference (32). However, the overall incidence was higher in men than women in our study. In multivariate Cox regression analysis, gender and site of tumor gradually became an independent risk factors for GI-NETs over time, while race might not be considered as an independent risk factor for GI-NETs. We might argue that gender differences emerge as the number of cases increases, while racial differences decrease in the context of the current global integration. This is good news for us, which can promote our further understanding of GI-NETs and improve the clinical management of patients.

Some studies have analyzed different sites of GI-NETs and reached conclusions (3), but no study has analyzed the overall epidemiological characteristics of GI-NETs at present., but no study has analyzed the overall epidemiological characteristics of GI-NETs at present. Here, our analysis of the epidemiology of GI-NETs from 1977 to 2016 may provide additional information about the disease to emphasize the urgency of early diagnosis and improved treatment of GI-NETs and help guide the development of clinical management programs.

There are some limitations in our study. First of all, the classification and definition of neuroendocrine tumors were not clear in the early stage, and most of them were benign lesions, which may result in the lack of certain information on unregistered GI-NETs in the SEER database. Deviations in data availability will have a certain impact on our results and conclusions. Secondly, some investigations have shown that the incidence of GI-NETs is related to other potential prognostic factors, such as marital status, but we did not include the analysis in this study.

## 5 Conclusion

Here, we collected eligible cases of GI-NETS from the U.S. Cancer Database from 1977 to 2016 for a new epidemiological analysis of the disease, including its incidence, survival, and risk factor assessment. In recent years, with the improvement of medical technology, the detection and treatment of GI-NETs have greatly helped, so the incidence and survival rate of GI-NETs has increased significantly. Age, stage, and pathological grade are considered independent risk factors for GI-NETs. According to our study, patients in the 60-74 age group, the small intestine group, the rectum group, and G1 patients had the highest incidence. The incidence is higher in men than women. The interaction between race and SES affects early diagnosis and treatment decisions.

## Data availability statement

The original contributions presented in the study are included in the article/**Supplementary Material**. Further inquiries can be directed to the corresponding author.

## Author contributions

PX, ML, and LW contributed to conception and design of the study. ML and MG organized the database. WL and SC performed the statistical analysis. YZ and ML wrote the first draft of the manuscript. ZG, RL, MG, and ML wrote sections of the manuscript. All authors contributed to the article and approved the submitted version.

## Supplementary material

Supplementary Figure 1 Trends in relative survival rate **(A–C)** and Kaplan–Meier survival curves **(D–G)** for patients with GI-NETs at 9 SEER sites according to SES group (low poverty, medium poverty, and high poverty) in 1977–1986, 1987–1996, 1997–2006, and 2007-2016.

Supplementary Figure 2 Trends in relative survival rate **(A–C)** and Kaplan–Meier survival curves **(D–G)** for patients with GI-NETs at 9 SEER sites according to grade group (G1, G1, and G3&4) in 1977–1986, 1987–1996, 1997–2006, and 2007-2016.

Supplementary Figure 3 Trends in relative survival rate **(A–C)** for patients with GI-NETs at 9 SEER sites according to site group (stomach, small intestine, appendix, colon, rectum) in 1977–1986, 1987–1996, 1997–2006, and 2007-2016.

Supplementary Figure 4 Kaplan–Meier survival curves **(A–E)** for patients with GI-NETs at 9 SEER sites according to site group (stomach, small intestine, appendix, colon, rectum) in 1977-2016, 1977–1986, 1987–1996, 1997–2006, and 2007-2016.
