# Supplementary material for: Trends in incidence and survival in patients with gastrointestinal neuroendocrine tumors: A SEER database analysis, 1977-2016
Source: Front Oncol. 2023 Jan 26;13:1079575. doi: 10.3389/fonc.2023.1079575 (PMC9909535; doi:10.3389/fonc.2023.1079575)
Supplement: Supplementary Figure 1 — Trends in relative survival rate (A–C) and Kaplan–Meier survival curves (D–G) for patients with GI-NETs at 9 SEER sites according to SES group (low poverty, medium poverty, and high poverty) in 1977–1986, 1987–1996, 1997–2006, and 2007-2016. [file DataSheet_1.zip › Data Sheet 1/Supplementary Table 1.docx]

**Supplementary Table 1.** The incidence of GI-NETs according to age group and decade within Sex, Race, SES, Grade, and Site groups from 1977 to 2016 at the nine original SEER sites. Data are incidence per 100,000 people by the year of diagnosis, with the number of patients in parentheses.

| Variable |  | Age group | Decade | | | |
| --- | --- | --- | --- | --- | --- | --- |
|  | Variable | Groups | 1977-1986 | 1987-1996 | 1997-2006 | 2007-2016 |
| Total |  |  |  |  |  |  |
|  |  | 0-75+ | 0.5(947) | 1.2(2557) | 2.1(5616) | 4(12863) |
|  |  | 0-44 | 0.1(141) | 0.2(380) | 0.4(706) | 1.0(1782) |
|  |  | 45-69 | 0.8(279) | 1.9(693) | 4.0(2068) | 8.1(5071) |
|  |  | 60-74 | 1.6(359) | 3.8(978) | 6.9(1822) | 11.5(4232) |
|  |  | 75+ | 1.8(168) | 4.1(506) | 6.7(1020) | 10.7(1778) |
| Sex | Male |  |  |  |  |  |
|  |  | 0-75+ | 0.5(441) | 1.3(1272) | 2.3(2804) | 4.2(6356) |
|  |  | 0-44 | 0.1(61) | 0.2(194) | 0.4(317) | 0.9(784) |
|  |  | 45-69 | 0.7(125) | 2.0(350) | 4.3(1090) | 8.2(2529) |
|  |  | 60-74 | 1.8(190) | 4.5(518) | 7.9(951) | 12.7(2201) |
|  |  | 75+ | 2.0(65) | 4.7(210) | 7.6(446) | 12.4(842) |
|  | Female |  |  |  |  |  |
|  |  | 0-75+ | 0.5(506) | 1.1(1285) | 2.0(2812) | 3.9(6507) |
|  |  | 0-44 | 0.1(80) | 0.2(186) | 0.4(389) | 1.2(998) |
|  |  | 45-69 | 0.9(154) | 1.9(343) | 3.7(978) | 8.0(2542) |
|  |  | 60-74 | 1.3(169) | 3.3(460) | 6.2(871) | 10.5(2031) |
|  |  | 75+ | 1.7(103) | 3.8(296) | 6.1(574) | 9.6(936) |
| Race | White |  |  |  |  |  |
|  |  | 0-75+ | 0.5(743) | 1.0(1813) | 1.8(3866) | 3.7(8987) |
|  |  | 0-44 | 0.1(101) | 0.2(242) | 0.4(473) | 1.0(1258) |
|  |  | 45-69 | 0.7(207) | 1.5(449) | 3.2(1337) | 6.9(3293) |
|  |  | 60-74 | 1.4(284) | 3.3(717) | 6.0(1281) | 10.5(3014) |
|  |  | 75+ | 1.8(151) | 3.8(405) | 6.0(775) | 10.5(1422) |
|  | Black |  |  |  |  |  |
|  |  | 0-75+ | 1.1(167) | 2.7(499) | 4.5(1087) | 6.5(2230) |
|  |  | 0-44 | 0.3(31) | 0.5(93) | 0.7(146) | 1.2(268) |
|  |  | 45-69 | 2.0(56) | 4.8(158) | 8.5(447) | 14.3(1039) |
|  |  | 60-74 | 3.8(64) | 8.7(170) | 15.6(344) | 20.5(722) |
|  |  | 75+ | 3.0(16) | 10.0(78) | 15.0(150) | 16.6(201) |
|  | Other |  |  |  |  |  |
|  |  | 0-75+ | 0.3(34) | 1.3(226) | 2.2(609) | 3.3(1345) |
|  |  | 0-44 | 0.1(7) | 0.3(37) | 0.4(77) | 0.7(185) |
|  |  | 45-69 | 0.7(15) | 2.6(81) | 4.7(254) | 7.4(579) |
|  |  | 60-74 | 0.9(11) | 4.1(86) | 6.7(184) | 10.2(440) |
|  |  | 75+ | 0.3(1) | 3.0(22) | 7.1(94) | 7.5(141) |
| SES | Low Poverty |  |  |  |  |  |
|  |  | 0-75+ | 0.5(490) | 1.0(1203) | 2.0(3176) | 4.0(7747) |
|  |  | 0-44 | 0.1(70) | 0.2(173) | 0.4(400) | 1.1(1142) |
|  |  | 45-69 | 0.8(143) | 1.6(328) | 3.9(1194) | 8.1(3071) |
|  |  | 60-74 | 1.6(193) | 3.2(451) | 6.5(982) | 11.3(2464) |
|  |  | 75+ | 1.7(84) | 3.7(251) | 6.6(600) | 10.6(1070) |
|  | Medium Poverty |  |  |  |  |  |
|  |  | 0-75+ | 0.5(428) | 1.4(1294) | 2.2(2289) | 4.0(4853) |
|  |  | 0-44 | 0.1(65) | 0.3(198) | 0.4(286) | 0.9(614) |
|  |  | 45-69 | 0.9(127) | 2.3(345) | 4.2(827) | 8.2(1893) |
|  |  | 60-74 | 1.6(157) | 4.6(503) | 7.7(783) | 12.1(1673) |
|  |  | 75+ | 2.0(79) | 4.9(248) | 6.7(393) | 11.0(673) |
|  | High Poverty |  |  |  |  |  |
|  |  | 0-75+ | 0.7(29) | 1.1(59) | 2.1(147) | 3.0(257) |
|  |  | 0-44 | 0.2(6) | 0.2(9) | 0.5(20) | 0.6(25) |
|  |  | 45-69 | 1.2(9) | 2.2(19) | 3.4(44) | 6.9(106) |
|  |  | 60-74 | 1.9(9) | 3.9(24) | 7.3(57) | 8.9(93) |
|  |  | 75+ | 2.7(5) | 2.5(7) | 7.0(26) | 7.1(33) |
| Grade | G1 |  |  |  |  |  |
|  |  | 0-75+ | 0.0(19) | 0.0(75) | 0.2(437) | 1.8(5891) |
|  |  | 0-44 | 0.0(4) | 0.0(12) | 0.0(53) | 0.5(953) |
|  |  | 45-69 | 0.0(6) | 0.1(23) | 0.3(167) | 3.7(2288) |
|  |  | 60-74 | 0.0(4) | 0.1(28) | 0.6(162) | 5.3(1942) |
|  |  | 75+ | 0.1(5) | 0.1(12) | 0.4(55) | 4.3(708) |
|  | G2 |  |  |  |  |  |
|  |  | 0-75+ | 0.0(5) | 0.0(53) | 0.1(191) | 0.4(1406) |
|  |  | 0-44 | 0.0(0) | 0.0(4) | 0.0(18) | 0.1(206) |
|  |  | 45-69 | 0.0(0) | 0.0(14) | 0.1(64) | 0.9(541) |
|  |  | 60-74 | 0.0(4) | 0.1(21) | 0.2(57) | 1.2(456) |
|  |  | 75+ | 0.0(1) | 0.1(14) | 0.3(52) | 1.2(203) |
|  | G3&4 |  |  |  |  |  |
|  |  | 0-75+ | 0.0(20) | 0.0(88) | 0.1(268) | 0.2(640) |
|  |  | 0-44 | 0.0(2) | 0.0(9) | 0.0(20) | 0.0(46) |
|  |  | 45-69 | 0.0(4) | 0.0(16) | 0.1(60) | 0.3(186) |
|  |  | 60-74 | 0.0(8) | 0.1(35) | 0.4(94) | 0.6(230) |
|  |  | 75+ | 0.1(6) | 0.2(28) | 0.6(94) | 1.0(178) |
| Site | stomach |  |  |  |  |  |
|  |  | 0-75+ | 0.0(54) | 0.1(227) | 0.2(649) | 0.4(1366) |
|  |  | 0-44 | 0.0(6) | 0.0(38) | 0.0(66) | 0.1(151) |
|  |  | 45-69 | 0.0(16) | 0.1(46) | 0.3(179) | 0.7(415) |
|  |  | 60-74 | 0.1(22) | 0.3(85) | 0.8(213) | 1.4(508) |
|  |  | 75+ | 0.1(10) | 0.5(58) | 1.2(191) | 1.8(292) |
|  | Small bowel |  |  |  |  |  |
|  |  | 0-75+ | 0.2(462) | 0.5(1037) | 0.7(1859) | 1.3(4259) |
|  |  | 0-44 | 0.0(39) | 0.1(91) | 0.1(143) | 0.2(298) |
|  |  | 45-69 | 0.4(136) | 0.7(244) | 1.1(558) | 2.0(1280) |
|  |  | 60-74 | 0.8(186) | 1.7(431) | 2.7(705) | 4.8(1734) |
|  |  | 75+ | 1.1(101) | 2.2(271) | 3.0(453) | 5.7(947) |
|  | appendix |  |  |  |  |  |
|  |  | 0-75+ | 0.1(118) | 0.1(153) | 0.1(368) | 0.5(1700) |
|  |  | 0-44 | 0.0(58) | 0.0(65) | 0.1(136) | 0.4(698) |
|  |  | 45-69 | 0.1(32) | 0.1(41) | 0.3(141) | 0.8(510) |
|  |  | 60-74 | 0.1(20) | 0.1(33) | 0.2(63) | 1.0(389) |
|  |  | 75+ | 0.1(8) | 0.1(14) | 0.2(28) | 0.6(103) |
|  | colon |  |  |  |  |  |
|  |  | 0-75+ | 0.1(153) | 0.2(353) | 0.3(737) | 0.4(1197) |
|  |  | 0-44 | 0.0(10) | 0.0(35) | 0.0(67) | 0.0(82) |
|  |  | 45-69 | 0.1(40) | 0.3(90) | 0.4(227) | 0.7(471) |
|  |  | 60-74 | 0.3(68) | 0.5(140) | 1.0(261) | 1.1(422) |
|  |  | 75+ | 0.4(35) | 0.7(88) | 1.2(182) | 1.3(222) |
|  | rectum |  |  |  |  |  |
|  |  | 0-75+ | 0.1(160) | 0.4(787) | 0.7(2003) | 1.3(4341) |
|  |  | 0-44 | 0.0(28) | 0.1(151) | 0.2(294) | 0.3(553) |
|  |  | 45-69 | 0.2(55) | 0.8(272) | 1.9(963) | 3.8(2395) |
|  |  | 60-74 | 0.3(63) | 1.1(289) | 2.2(580) | 3.1(1179) |
|  |  | 75+ | 0.1(14) | 0.6(75) | 1.1(166) | 1.3(214) |

Abbreviation: SES, socioeconomic status.
