# Supplementary material for: Trends in incidence and survival in patients with gastrointestinal neuroendocrine tumors: A SEER database analysis, 1977-2016
Source: Front Oncol. 2023 Jan 26;13:1079575. doi: 10.3389/fonc.2023.1079575 (PMC9909535; doi:10.3389/fonc.2023.1079575)
Supplement: Supplementary Figure 1 — Trends in relative survival rate (A–C) and Kaplan–Meier survival curves (D–G) for patients with GI-NETs at 9 SEER sites according to SES group (low poverty, medium poverty, and high poverty) in 1977–1986, 1987–1996, 1997–2006, and 2007-2016. [file DataSheet_1.zip › Data Sheet 1/Supplementary Table 2 .docx]

**Supplementary Table 2.** 12-month, 60-month, and 120-month relative survival rates of GI-NETs patients according to sex, age group, and calendar period from 1977 to 2016 at nine SEER sites. Data are means ± standard error of the mean, with the number of patients in parentheses.

|  |  | Sex | |
| --- | --- | --- | --- |
| Decade | Age Group | Male | Female |
| 77-86 | 12-Mo RS |  |  |
|  | All | 83.3±2.1(374) | 84.5±1.9(427) |
|  | 0-44 | 93.6±3.2(60) | 97.3±1.9(73) |
|  | 45-59 | 86.0±3.3(115) | 87.7±2.8(140) |
|  | 60-74 | 81.2±3.4(154) | 85.2±3.3(134) |
|  | 75+ | 68.7±8.0(45) | 65.4±5.8(80) |
|  | 60-Mo RS |  |  |
|  | All | 67.1±2.9(374) | 72.1±2.5(427) |
|  | 0-44 | 90.9±3.9(60) | 96.1±2.4(73) |
|  | 45-59 | 68.3±4.7(115) | 79.0±3.7(140) |
|  | 60-74 | 62.2±4.8(154) | 64.5±4.7(134) |
|  | 75+ | 42.1±10.4(45) | 47.6±7.7(80) |
|  | 120-Mo RS |  |  |
|  | All | 62.6±3.4(374) | 61.8±2.9(427) |
|  | 0-44 | 90.9±3.9(60) | 84.7±4.5(73) |
|  | 45-59 | 66.4±5.3(115) | 70.4±4.4(140) |
|  | 60-74 | 49.8±5.7(154) | 52.7±5.5(134) |
|  | 75+ | 39.9±11.1(45) | 31.8±9.1(80) |
| 87-96 | 12-Mo RS |  |  |
|  | All | 89.4±1.1(1047) | 89.5±1.0(1058) |
|  | 0-44 | 95.0±1.6(189) | 93.7±1.9(174) |
|  | 45-59 | 94.0±1.4(320) | 95.6±1.2(304) |
|  | 60-74 | 88.2±1.8(398) | 90.0±1.7(354) |
|  | 75+ | 74.4±4.3(140) | 77.0±3.1(226) |
|  | 60-Mo RS |  |  |
|  | All | 80.5±1.6(1047) | 80.0±1.6(1058) |
|  | 0-44 | 91.4±2.2(189) | 90.7±2.3(174) |
|  | 45-59 | 89.2±2.1(320) | 87.3±2.1(304) |
|  | 60-74 | 76.2±2.8(398) | 74.4±2.8(354) |
|  | 75+ | 51.1±6.4(140) | 69.5±4.9(226) |
|  | 120-Mo RS |  |  |
|  | All | 74.2±2.0(1047) | 70.0±1.9(1058) |
|  | 0-44 | 88.3±2.7(189) | 89.5±2.5(174) |
|  | 45-59 | 82.9±2.8(320) | 79.4±2.7(304) |
|  | 60-74 | 66.4±3.6(398) | 60.8±3.4(354) |
|  | 75+ | 48.6±9.2(140) | 47.7±6.6(226) |
| 97-06 | 12-Mo RS |  |  |
|  | All | 91.8±0.6(2284) | 92.9±0.6(2339) |
|  | 0-44 | 96.7±1.1(312) | 97.6±0.8(363) |
|  | 45-59 | 95.2±0.7(989) | 97.0±0.6(857) |
|  | 60-74 | 90.2±1.2(720) | 92.7±1.1(705) |
|  | 75+ | 77.4±3.0(263) | 80.2±2.2(414) |
|  | 60-Mo RS |  |  |
|  | All | 85.6±1.0(1047) | 86.2±0.9(2339) |
|  | 0-44 | 91.3±1.7(312) | 93.7±1.3(363) |
|  | 45-59 | 89.9±1.1(989) | 91.2±1.1(857) |
|  | 60-74 | 83.1±1.9(720) | 84.6±1.7(705) |
|  | 75+ | 67.2±4.7(263) | 71.2±3.2(414) |
|  | 120-Mo RS |  |  |
|  | All | 80.8±1.2(1047) | 80.7±1.2(2339) |
|  | 0-44 | 87.0±2.1(312) | 92.1±1.6(363) |
|  | 45-59 | 86.0±1.5(989) | 86.6±1.4(857) |
|  | 60-74 | 78.4±2.5(720) | 76.8±2.3(705) |
|  | 75+ | 52.3±6.5(263) | 61.0±4.9(414) |
| 07-16 | 12-Mo RS |  |  |
|  | All | 94.8±0.4(5134) | 95.7±0.3(5288) |
|  | 0-44 | 97.8±0.6(740) | 99.1±0.3(925) |
|  | 45-59 | 96.6±0.4(2271) | 97.9±0.3(2194) |
|  | 60-74 | 94.7±0.6(1634) | 94.9±0.6(1522) |
|  | 75+ | 82.7±2.0(489) | 85.1±1.6(647) |
|  | 60-Mo RS |  |  |
|  | All | 89.8±0.6(5134) | 90.4±0.6(5288) |
|  | 0-44 | 94.8±1.0(740) | 97.2±0.7(925) |
|  | 45-59 | 92.9±0.7(2271) | 93.7±0.6(2194) |
|  | 60-74 | 87.9±1.2(1634) | 87.4±1.2(1522) |
|  | 75+ | 72.2±3.6(489) | 75.9±2.9(647) |
|  | 120-Mo RS |  |  |
|  | All | 85.9±1.0(5134) | 86.6±1.0(5288) |
|  | 0-44 | 92.3±1.6(740) | 93.3±1.5(925) |
|  | 45-59 | 90.5±1.3(2271) | 90.7±1.1(2194) |
|  | 60-74 | 82.1±2.1(1634) | 80.9±2.1(1522) |
|  | 75+ | 56.4±7.4(489) | 73.3±5.0(647)*** |

Abbreviations: Mo, month; RS, relative survival; SEM, standard error of the mean.

**p* < 0.01, ***p* < 0.001, and ****p* < 0.0001
