# Supplementary material for: Trends in incidence and survival in patients with gastrointestinal neuroendocrine tumors: A SEER database analysis, 1977-2016
Source: Front Oncol. 2023 Jan 26;13:1079575. doi: 10.3389/fonc.2023.1079575 (PMC9909535; doi:10.3389/fonc.2023.1079575)
Supplement: Supplementary Figure 1 — Trends in relative survival rate (A–C) and Kaplan–Meier survival curves (D–G) for patients with GI-NETs at 9 SEER sites according to SES group (low poverty, medium poverty, and high poverty) in 1977–1986, 1987–1996, 1997–2006, and 2007-2016. [file DataSheet_1.zip › Data Sheet 1/Supplementary Table 3 .docx]

**Supplementary Table 3.**12-month, 60-month, and 120-month relative survival rates of GI-NETs patients according to race, age group, and calendar period from 1977 to 2016 at nine SEER sites. Data are means ± standard error of the mean, with the number of patients in parentheses.

|  |  | Race | | |
| --- | --- | --- | --- | --- |
| Decade | Age Group | White | Black | Other |
| 77-86 | 12-Mo RS |  |  |  |
|  | All | 84.9±1.5(624) | 78.6±3.6(143) | 87.7±6.1(31) |
|  | 0-44 | 97.9±1.5(94) | 87.4±6.0(31) | 100.0±0.0(6) |
|  | 45-59 | 88.4±2.4(187) | 79.8±5.7(52) | 93.7±6.5(15) |
|  | 60-74 | 85.1±2.5(229) | 74.2±6.5(50) | 78.0±13.9(9) |
|  | 75+ | 67.6±4.9(114) | 64.0±16.5(10) | 0.0±0.0(1) |
|  | 60-Mo RS |  |  |  |
|  | All | 69.3±2.2(624) | 68.1±4.5(143) | 81.9±7.2(31) |
|  | 0-44 | 95.1±2.3(94) | 87.4±6.0(31) | 100.0±0.0(6) |
|  | 45-59 | 73.7±3.5(187) | 71.8±6.9(52) | 87.4±8.9(15) |
|  | 60-74 | 64.7±3.8(229) | 54.8±8.4(50) | 67.9±16.0(9) |
|  | 75+ | 47.4±6.7(114) | 35.6±17.2(10) | 0.0±0.0(1) |
|  | 120-Mo RS |  |  |  |
|  | All | 62.8±2.6(624) | 58.8±5.2(143) | 66.2±9.4(31) |
|  | 0-44 | 88.8±3.5(94) | 87.4±6.0(31) | 84.1±15.4(6) |
|  | 45-59 | 69.2±3.9(187) | 66.2±8.2(52) | 70.0±12.8(15) |
|  | 60-74 | 53.6±4.4(229) | 37.1±8.9(50) | 49.6±18.5(9) |
|  | 75+ | 43.6±9.2(114) | 0.0±0.0(10) | 0.0±0.0(1) |
| 87-96 | 12-Mo RS |  |  |  |
|  | All | 89.1±0.9(1482) | 88.6±1.7(414) | 93.3±1.9(196) |
|  | 0-44 | 95.0±1.5(231) | 92.6±2.8(92) | 94.2±4.0(34) |
|  | 45-59 | 94.6±1.2(404) | 93.5±2.2(144) | 97.6±1.9(72) |
|  | 60-74 | 89.3±1.4(554) | 85.9±3.5(123) | 91.1±3.5(72) |
|  | 75+ | 76.0±2.8(293) | 73.4±6.6(55) | 80.4±10.1(18) |
|  | 60-Mo RS |  |  |  |
|  | All | 79.6±1.3(1482) | 78.5±2.6(414) | 86.7±2.9(196) |
|  | 0-44 | 90.8±2.0(231) | 89.7±3.5(92) | 94.2±4.0(34) |
|  | 45-59 | 88.0±1.8(404) | 86.5±3.5(144) | 91.9±3.6(72) |
|  | 60-74 | 75.4±2.3(554) | 69.3±5.3(123) | 82.0±5.4(72) |
|  | 75+ | 64.6±4.5(293) | 53.6±9.9(55) | 64.9±15.3(18) |
|  | 120-Mo RS |  |  |  |
|  | All | 70.3±1.7(1482) | 70.8±3.2(414) | 83.5±3.6(196) |
|  | 0-44 | 90.2±2.2(231) | 82.4±4.6(92) | 94.2±4.0(34) |
|  | 45-59 | 79.9±2.4(404) | 79.6±4.3(144) | 89.0±4.5(72) |
|  | 60-74 | 62.0±2.9(554) | 62.9±6.4(123) | 72.7±6.8(72) |
|  | 75+ | 48.9±5.8(293) | 26.3±11.2  (55)* | 64.9±15.3(18) |
| 97-06 | 12-Mo RS |  |  |  |
|  | All | 91.9±0.5(3141) | 91.4±1.0(904) | 96.0±0.9(527) |
|  | 0-44 | 98.2±0.7(454) | 93.0±2.2(138) | 98.7±1.3(74) |
|  | 45-59 | 96.0±0.6(1191) | 94.8±1.2(393) | 98.1±1.0(232) |
|  | 60-74 | 91.1±1.0(986) | 89.7±2.1(270) | 95.6±1.8(157) |
|  | 75+ | 78.0±2.1(510) | 80.4±4.6(103) | 84.5±4.9(64) |
|  | 60-Mo RS |  |  |  |
|  | All | 85.8±0.8(3141) | 83.6±1.6(904) | 88.5±1.7(527) |
|  | 0-44 | 94.8±1.1(454) | 85.2±3.2(138) | 92.3±3.2(74) |
|  | 45-59 | 90.2±1.0(1191) | 88.9±1.9(393) | 93.1±1.9(232) |
|  | 60-74 | 84.1±1.5(986) | 77.9±3.3(270) | 88.9±2.9(157) |
|  | 75+ | 69.9±3.2(510) | 72.6±6.8(103) | 62.6±7.5(64) |
|  | 120-Mo RS |  |  |  |
|  | All | 79.9±1.0(3141) | 79.1±2.0(904) | 84.9±2.1(527) |
|  | 0-44 | 91.9±1.4(454) | 82.5±3.5(138) | 89.0±3.9(74) |
|  | 45-59 | 85.5±1.3(1191) | 83.0±2.5(393) | 91.7±2.2(232) |
|  | 60-74 | 77.1±2.0(986) | 73.7±4.1(270) | 82.4±4.3(157) |
|  | 75+ | 55.1±4.6(510) | 70.9±7.6(103)  (103)* | 55.2±10.0(64) |
| 07-16 | 12-Mo RS |  |  |  |
|  | All | 94.7±0.3(7208) | 96.2±0.5(1813) | 96.4±0.6(1117) |
|  | 0-44 | 98.6±0.4(1184) | 98.5±0.8(245) | 97.6±1.2(168) |
|  | 45-59 | 96.7±0.4(2898) | 97.2±0.6(916) | 98.8±0.5(501) |
|  | 60-74 | 94.1±0.6(2231) | 95.4±1.1(527) | 97.6±1.0(345) |
|  | 75+ | 84.1±1.4(895) | 85.9±3.7(125) | 78.5±4.3(103) |
|  | 60-Mo RS |  |  |  |
|  | All | 89.0±0.5(7208) | 91.0±1.0(1813) | 92.3±1.1(1117) |
|  | 0-44 | 96.1±0.7(1184) | 95.7±1.7(245) | 95.8±1.6(168) |
|  | 45-59 | 92.3±0.6(2898) | 92.9±1.1(916) | 96.7±1.0(501) |
|  | 60-74 | 86.2±1.0(2231) | 88.8±1.9(527) | 92.4±2.1(345) |
|  | 75+ | 75.3±2.6(895) | 73.3±6.7(125) | 62.0±6.7(103) |
|  | 120-Mo RS |  |  |  |
|  | All | 85.1±0.9(7208) | 85.9±1.7(1813) | 88.2±1.9(1117) |
|  | 0-44 | 94.5±1.1(1184) | 86.5±3.7(245) | 90.4±3.6(168) |
|  | 45-59 | 89.5±1.0(2898) | 89.9±1.9(916) | 93.6±2.3(501) |
|  | 60-74 | 79.2±1.8(2231) | 84.4±3.2(527) | 85.4±3.7(345) |
|  | 75+ | 67.5±4.4(895) | 46.2±13.6  (125)*** | 57.3±11.0(103) |

Abbreviations: Mo, month; RS, relative survival; SEM, standard error of the mean.

**p* < 0.01, ***p* < 0.001, and ****p* < 0.0001 for comparisons with the preceding group.
