# Supplementary material for: Trends in incidence and survival in patients with gastrointestinal neuroendocrine tumors: A SEER database analysis, 1977-2016
Source: Front Oncol. 2023 Jan 26;13:1079575. doi: 10.3389/fonc.2023.1079575 (PMC9909535; doi:10.3389/fonc.2023.1079575)
Supplement: Supplementary Figure 1 — Trends in relative survival rate (A–C) and Kaplan–Meier survival curves (D–G) for patients with GI-NETs at 9 SEER sites according to SES group (low poverty, medium poverty, and high poverty) in 1977–1986, 1987–1996, 1997–2006, and 2007-2016. [file DataSheet_1.zip › Data Sheet 1/Supplementary Table 4 .docx]

**Supplementary Table 4.**12-month, 60-month, and 120-month relative survival rates of GI-NETs patients according to SES, age group, and calendar period from 1977 to 2016 at nine SEER sites. Data are means ± standard error of the mean, with the number of patients in parentheses.

|  |  | SES | | |
| --- | --- | --- | --- | --- |
| Decade | Age Group | Low Poverty | Medium Poverty | High Poverty |
| 77-86 | 12-Mo RS |  |  |  |
|  | All | 86.4±1.8(410) | 81.9±2.1(368) | 70.5±9.7(23) |
|  | 0-44 | 98.6±1.6(65) | 92.3±3.4(64) | 100.0±0.0(4) |
|  | 45-59 | 89.7±2.8(128) | 85.5±3.3(118) | 67.0±15.8(9) |
|  | 60-74 | 84.1±3.1(156) | 81.4±3.7(125) | 87.4±13.5(7) |
|  | 75+ | 72.3±6.5(61) | 64.1±6.8(61) | 0.0±0.0(3) |
|  | 60-Mo RS |  |  |  |
|  | All | 71.2±2.6(410) | 68.2±2.9(368) | 67.1±10.2(23) |
|  | 0-44 | 97.5±2.2(65) | 89.5±3.9(64) | 100.0±0.0(4) |
|  | 45-59 | 75.2±4.2(128) | 74.0±4.4(118) | 67.0±15.8(9) |
|  | 60-74 | 63.8±4.5(156) | 61.2±5.2(125) | 75.8±18.1(7) |
|  | 75+ | 50.6±8.9(61) | 41.3±8.2(61) | 0.0±0.0(3) |
|  | 120-Mo RS |  |  |  |
|  | All | 64.9±3.0(410) | 59.4±3.3(368) | 52.2±12.4(23) |
|  | 0-44 | 92.5±3.7(65) | 83.5±5.0(64) | 100.0±0.0(4) |
|  | 45-59 | 73.1±4.4(128) | 64.9±5.1(118) | 36.2±17.1(9) |
|  | 60-74 | 53.3±5.3(156) | 46.4±5.9(125) | 58.2±25.4(7) |
|  | 75+ | 33.5±11.0(61) | 40.6±9.7(61) | 0.0±0.0(3) |
| 87-96 | 12-Mo RS |  |  |  |
|  | All | 89.9±1.0(1006) | 88.8±1.1(1048) | 92.9±3.9(50) |
|  | 0-44 | 95.8±1.6(161) | 93.9±1.7(193) | 77.9±13.9(9) |
|  | 45-59 | 94.2±1.4(296) | 94.9±1.3(309) | 100.0±0.0(18) |
|  | 60-74 | 90.1±1.7(362) | 87.5±1.9(370) | 96.6±5.0(20) |
|  | 75+ | 75.7±3.4(187) | 60.9±3.5(176) | 67.1±27.4(3) |
|  | 60-Mo RS |  |  |  |
|  | All | 81.7±1.6(1006) | 78.9±1.6 (1048) | 81.7±6.5(50) |
|  | 0-44 | 92.8±2.1(161) | 89.7±2.3(193) | 77.9±13.9 (9) |
|  | 45-59 | 87.4±2.2(296) | 88.6±2.1(309) | 96.7±5.5(18) |
|  | 60-74 | 77.4±2.8(362) | 73.3±2.9(370) | 74.2±1.2(20) |
|  | 75+ | 67.7±5.2(187) | 57.8±5.7(176) | 35.6±29.0(3) |
|  | 120-Mo RS |  |  |  |
|  | All | 74.2±2.0(1006) | 69.8±2.0(1048) | 71.1±7.9(50) |
|  | 0-44 | 92.8±2.1(161) | 85.9±2.8(193) | 77.9±13.9 (9) |
|  | 45-59 | 81.9±2.8(296) | 80.3±2.8(309) | 80.9±10.2(18) |
|  | 60-74 | 66.4±3.6(362) | 60.9±3.5(370) | 65.4±11.2(20) |
|  | 75+ | 51.3±7.3(187) | 42.5±7.5(176) | 0.0±0.0(3) |
| 97-06 | 12-Mo RS |  |  |  |
|  | All | 93.4±0.5(2615) | 91.0±0.7(1885) | 90.3±2.9(119) |
|  | 0-44 | 99.6±0.4(381) | 93.6±1.5(276) | 100.0±0.0(18) |
|  | 45-59 | 97.0±0.6(1073) | 95.2±0.8(731) | 87.4±5.4(39) |
|  | 60-74 | 91.7±1.1(758) | 91.1±1.3(621) | 92.6±4.2(46) |
|  | 75+ | 80.8±2.3(403) | 76.3±3.0(257) | 79.6±11.5(16) |
|  | 60-Mo RS |  |  |  |
|  | All | 91.8±0.9(2615) | 83.8±1.1  (1885)*** | 83.0±4.1(119) |
|  | 0-44 | 95.3±1.2(381) | 88.8±2.0(276) | 94.5±5.7(18) |
|  | 45-59 | 91.3±1.01073) | 89.8±1.3(731) | 77.7±7.0(39) |
|  | 60-74 | 85.6±1.7(758) | 81.6±2.0(621) | 83.1±6.5(46) |
|  | 75+ | 71.8±3.3(403) | 64.6±4.6(257) | 74.8±12.6(16) |
|  | 120-Mo RS |  |  |  |
|  | All | 82.1±1.1(2615) | 78.7±1.3(1885) | 77.1±5.8(119) |
|  | 0-44 | 92.1±1.6(381) | 86.7±2.2(276) | 89.6±7.9(18) |
|  | 45-59 | 87.6±1.31073) | 84.7±1.7(731) | 68.9±8.7(39) |
|  | 60-74 | 79.2±2.3(758) | 75.3±2.6(621) | 80.1±9.4(46) |
|  | 75+ | 56.7±5.0(403) | 55.5±6.1(257) | 67.3±29.2(16) |
| 07-16 | 12-Mo RS |  |  |  |
|  | All | 95.9±0.3(6284) | 94.3±0.4(3917) | 93.6±1.8(218) |
|  | 0-44 | 98.8±0.4(1063) | 98.4±0.6(577) | 90.6±6.4(24) |
|  | 45-59 | 98.0±0.3(2697) | 96.0±0.5(1674) | 93.8±2.6(94) |
|  | 60-74 | 94.8±0.6(1838) | 94.7±0.7(1238) | 96.6±2.2(79) |
|  | 75+ | 85.9±1.6(686) | 81.3±2.2(428) | 79.1±9.7(21) |
|  | 60-Mo RS |  |  |  |
|  | All | 91.0±0.5(6284) | 88.9±0.7(3917) | 86.5±3.1(218) |
|  | 0-44 | 96.0±0.8(1063) | 96.4±0.9(577) | 90.6±6.4(24) |
|  | 45-59 | 94.6±0.6(2697) | 91.7±0.8(1674) | 83.9±4.4(94) |
|  | 60-74 | 87.6±1.1(1838) | 87.6±1.3(1238) | 88.8±5.0(79) |
|  | 75+ | 76.6±3.0(686) | 70.4±3.6(428) | 75.3±13.1(21) |
|  | 120-Mo RS |  |  |  |
|  | All | 87.6±0.9(6284) | 83.9±1.2  (3917)** | 84.9±3.8(218) |
|  | 0-44 | 93.0±1.3(1063) | 92.6±2.0(577) | 90.6±6.4(24) |
|  | 45-59 | 92.0±1.0(2697) | 88.9±1.4(1674) | 82.4±5.1(94) |
|  | 60-74 | 82.2±1.9(1838) | 80.1±2.2(1238) | 76.9±9.5(79) |
|  | 75+ | 70.4±5.1(686) | 55.2±7.0  (428)*** | 75.3±13.1(21) |

Abbreviations: Mo, month; RS, relative survival; SEM, standard error of the mean.

*P < 0.01, **P < 0.001, and ***P < 0.0001 for comparisons with the Low Poverty group.
