# Supplementary material for: Trends in incidence and survival in patients with gastrointestinal neuroendocrine tumors: A SEER database analysis, 1977-2016
Source: Front Oncol. 2023 Jan 26;13:1079575. doi: 10.3389/fonc.2023.1079575 (PMC9909535; doi:10.3389/fonc.2023.1079575)
Supplement: Supplementary Figure 1 — Trends in relative survival rate (A–C) and Kaplan–Meier survival curves (D–G) for patients with GI-NETs at 9 SEER sites according to SES group (low poverty, medium poverty, and high poverty) in 1977–1986, 1987–1996, 1997–2006, and 2007-2016. [file DataSheet_1.zip › Data Sheet 1/Supplementary Table 5 .docx]

**Supplementary Table 5.**12-month, 60-month, and 120-month relative survival rates of GI-NETs patients according to grade, age group, and calendar period from 1977 to 2016 at nine SEER sites. Data are means ± standard error of the mean, with the number of patients in parentheses.

|  |  | Grade | | |
| --- | --- | --- | --- | --- |
| Decade | Age Group | Grade1 | Grade2 | Grade3&4 |
| 77-86 | 12-Mo RS |  |  |  |
|  | All | 72.8±11.4(17) | 77.2±22.3(4) | 27.2±11.6(15) |
|  | 0-44 | 75.0±21.7(4) | 0.0±0.0(0) | 100.0±0.0(2) |
|  | 45-59 | 100.0±0.0(6) | 0.0±0.0(0) | 33.5±27.4(3) |
|  | 60-74 | 51.1±25.5(4) | 77.2±22.3(4) | 17.1±15.6(6) |
|  | 75+ | 35.9±32.2(3) | 0.0±0.0(0) | 0.0±0.0(4) |
|  | 60-Mo RS |  |  |  |
|  | All | 72.8±11.4(17) | 51.6±25.8(4) | 13.7±9.0(15) |
|  | 0-44 | 75.0±21.7(4) | 0.0±0.0(0) | 50.2±35.5(2) |
|  | 45-59 | 100.0±0.0(6) | 0.0±0.0(0) | 0.0±0.0(3) |
|  | 60-74 | 27.6±23.9(4) | 51.6±25.8(4) | 17.1±15.6(6) |
|  | 75+ | 0.0±0.0(3) | 0.0±0.0(0) | 0.0±0.0(4) |
|  | 120-Mo RS |  |  |  |
|  | All | 72.8±11.4(17) | 32.3±28.0(4) | 13.7±9.0(15) |
|  | 0-44 | 75.0±21.7(4) | 0.0±0.0(0) | 50.2±35.5(2) |
|  | 45-59 | 100.0±0.0(6) | 0.0±0.0(0) | 0.0±0.0(3) |
|  | 60-74 | 27.6±23.9(4) | 32.3±28.0(4) | 17.1±15.6(6) |
|  | 75+ | 0.0±0.0(3) | 0.0±0.0(0) | 0.0±0.0(4) |
| 87-96 | 12-Mo RS |  |  |  |
|  | All | 92.7±3.7(65) | 80.3±6.5(42) | 45.7±6.1(70)** |
|  | 0-44 | 100.0±0.0(12) | 75.1±21.7(4) | 50.1±17.7(8) |
|  | 45-59 | 95.9±4.7(21) | 92.8±7.4(13) | 54.3±13.9(13) |
|  | 60-74 | 92.1±6.5(21) | 65.2±13.0(14) | 49.7±9.5(29) |
|  | 75+ | 74.6±13.8(11) | 84.2±12.0(11) | 32.2±11.0(20) |
|  | 60-Mo RS |  |  |  |
|  | All | 77.9±6.4(65) | 55.5±8.6(42) | 26.7±5.9(70)** |
|  | 0-44 | 84.0±10.8(12) | 75.1±21.7(4) | 12.5±11.7(8) |
|  | 45-59 | 83.7±8.9(21) | 71.2±13.2(13) | 31.7±13.2(13) |
|  | 60-74 | 75.8±11.7(21) | 31.0±13.1(14) | 31.5±9.5(29) |
|  | 75+ | 52.9±21.1(11) | 57.6±19.0(11) | 20.2±10.7(20) |
|  | 120-Mo RS |  |  |  |
|  | All | 71.6±7.7(65) | 50.1±9.9(42) | 17.6±5.5(70)* |
|  | 0-44 | 84.0±10.8(12) | 75.1±21.7(4) | 12.7±11.7(8) |
|  | 45-59 | 80.4±9.8(21) | 64.2±14.1(13) | 17.0±11.1(13) |
|  | 60-74 | 70.4±14.6(21) | 31.0±13.1(14) | 23.3±9.5(29) |
|  | 75+ | 0.0±0.0(11) | 22.8±21.8(11) | 8.9±8.6(20) |
| 97-06 | 12-Mo RS |  |  |  |
|  | All | 96.0±1.2(374) | 85.9±3.1(153) | 47.6±3.6  (206)*** |
|  | 0-44 | 100.0±0.0(51) | 100.0±0.0(15) | 68.5±10.7(19) |
|  | 45-59 | 98.9±0.9(152) | 91.5±3.9(56) | 50.3±6.8(54)*** |
|  | 60-74 | 96.3±1.9(130) | 83.4±5.9(44) | 44.7±5.9(74)*** |
|  | 75+ | 76.6±7.4(41) | 73.6±8.1(48) | 41.8±6.8(59)* |
|  | 60-Mo RS |  |  |  |
|  | All | 90.2±2.1(374) | 64.6±4.6  (153) *** | 25.4±3.3  (206)*** |
|  | 0-44 | 96.7±2.8(51) | 74.0±11.5(15) | 37.1±11.1(19) |
|  | 45-59 | 92.9±2.5(152)* | 67.8±6.6(56) | 24.6±6.1(54)*** |
|  | 60-74 | 89.3±3.8(130) | 67.7±8.2(44) | 22.4±5.1(74)*** |
|  | 75+ | 72.3±8.4(41) | 49.6±11.2(48) | 25.0±6.8(59) |
|  | 120-Mo RS |  |  |  |
|  | All | 82.2±2.9(374) | 56.7±5.2(153)*** | 19.9±3.4(206)*** |
|  | 0-44 | 85.9±5.3(51) | 67.6±12.3(15) | 37.1±11.1(19) |
|  | 45-59 | 88.5±3.4(152) | 58.5±7.2(56)** | 19.0±5.7(54)*** |
|  | 60-74 | 76.5±5.2(130) | 65.0±8.8(44) | 15.4±4.8(74)*** |
|  | 75+ | 61.9±14.9(41)** | 25.1±13.8(48) | 20.6±8.6(59) |
| 07-16 | 12-Mo RS |  |  |  |
|  | All | 98.5±0.2(4836) | 96.7±0.6(1109) | 55.9±2.3(497)*** |
|  | 0-44 | 99.8±0.2(898) | 100.0±0.0(195) | 61.6±7.6(42)* |
|  | 45-59 | 99.5±0.2(2008) | 96.8±0.9(471) | 60.7±3.9(162)*** |
|  | 60-74 | 98.2±0.4(1479) | 97.3±1.1(318) | 56.9±3.7(183)*** |
|  | 75+ | 92.0±1.6(451) | 88.4±3.4(125) | 44.9±5.0(110)*** |
|  | 60-Mo RS |  |  |  |
|  | All | 95.5±0.6(4836) | 88.5±1.4(1109)* | 29.9±2.3(497)*** |
|  | 0-44 | 98.9±0.6(898) | 96.5±1.8(195) | 40.4±8.1(42)*** |
|  | 45-59 | 96.9±0.6(2008) | 91.4±1.7(471) | 33.5±3.9(162)*** |
|  | 60-74 | 94.5±1.1(1479) | 84.9±2.8(318) | 33.6±3.8(183)*** |
|  | 75+ | 86.0±3.8(451) | 71.5±5.2(125) | 14.0±4.2(110)*** |
|  | 120-Mo RS |  |  |  |
|  | All | 91.0±1.6(4836) | 79.9±3.1(1109)*** | 27.1±2.6(497)*** |
|  | 0-44 | 95.2±2.0(898) | 92.3±3.7(195) | 0.0±0.0(42)*** |
|  | 45-59 | 92.9±1.8(2008) | 82.6±4.5(471)* | 31.7±4.0(162)*** |
|  | 60-74 | 89.8±2.2(1479) | 72.9±5.6(318)*** | 25.6±4.5(183)*** |
|  | 75+ | 68.6±11.1(451) | 55.0±11.8(125) | 14.0±4.2(110)*** |

Abbreviations: Mo, month; RS, relative survival; SEM, standard error of the mean.

**p* < 0.05, ***p* < 0.001, and ****p* < 0.0001 for comparisons with the former grade group.
