# Supplementary material for: Trends in incidence and survival in patients with gastrointestinal neuroendocrine tumors: A SEER database analysis, 1977-2016
Source: Front Oncol. 2023 Jan 26;13:1079575. doi: 10.3389/fonc.2023.1079575 (PMC9909535; doi:10.3389/fonc.2023.1079575)
Supplement: Supplementary Figure 1 — Trends in relative survival rate (A–C) and Kaplan–Meier survival curves (D–G) for patients with GI-NETs at 9 SEER sites according to SES group (low poverty, medium poverty, and high poverty) in 1977–1986, 1987–1996, 1997–2006, and 2007-2016. [file DataSheet_1.zip › Data Sheet 1/Supplementary Table 6.docx]

**Supplementary Table 6.** 12-month, 60-month, and 120-month relative survival rates of GI-NETs patients according to Site, age group, and calendar period from 1977 to 2016 at nine SEER sites. Data are means ± standard error of the mean, with the number of patients in parentheses.

|  |  | Site | | | | |
| --- | --- | --- | --- | --- | --- | --- |
| Decade | Age Group | Stomach | Small bowel | Appendix | Colon | Rectum |
| 77-86 | 12-Mo RS |  |  |  |  |  |
|  | All | 82.9±5.7(45) | 88.3±1.8(382) | 91.6±2.9(99) | 63.3±4.4(128) | 85.1±3.1(147) |
|  | 0-44 | 100.0±0.0(5) | 94.7±3.8(37) | 98.2±1.8(54) | 80.0±12.7(10) | 96.5±3.6(27) |
|  | 45-59 | 81.5±9.8(16) | 91.7±2.6(121) | 87.4±7.4(25) | 75.5±6.9(40) | 87.4±4.7(53) |
|  | 60-74 | 94.6±5.7(17) | 88.8±2.9(148) | 88.7±8.4(16) | 63.2±6.9(52) | 80.0±5.7(55) |
|  | 75+ | 43.7±19.1(7) | 78.2±5.5(76) | 52.0±26.0(4) | 37.4±10.1(26) | 70.8±14.4(12) |
|  | 60-Mo RS |  |  |  |  |  |
|  | All | 67.9±7.9(45) | 69.3±2.9(382) | 86.8±3.7(99) | 47.3±4.9(128) | 78.4±3.8(147) |
|  | 0-44 | 100.0±0.0(5) | 92.2±4.6(37) | 96.7±2.6(54) | 70.2±14.5(10) | 96.5±3.6(27 |
|  | 45-59 | 69.7±11.7(16) | 74.1±4.4(121) | 77.4±8.7(25) | 56.9±8.1(40) | 86.5±5.4(53) |
|  | 60-74 | 66.0±13.4(17) | 65.0±4.7(148) | 83.3±10.0(16) | 44.5±7.8(52) | 67.2±7.1(55) |
|  | 75+ | 32.2±19.2(7) | 56.5±8.8(76) | 0.0±0.0(4) | 24.9±10.0(26) | 41.5±16.9(12) |
|  | 120-Mo RS |  |  |  |  |  |
|  | All | 60.1±9.2(45) | 58.0±3.4(382) | 85.0±4.4(99) | 37.6±5.0(128) | 76.2±4.7(147) |
|  | 0-44 | 100.0±0.0(5) | 82.2±6.7(37) | 92.3±4.0(54) | 61.0±15.8(10) | 95.0±5.2(27) |
|  | 45-59 | 66.3±12.8(16) | 67.5±5.1(121) | 77.4±8.7(25) | 46.3±8.5(40) | 81.2±6.7(53) |
|  | 60-74 | 47.2±15.5(17) | 47.1±5.4(148) | 78.6±15.2(16) | 31.7±7.6(52) | 66.2±9.1(55) |
|  | 75+ | 29.0±26.9(7) | 44.3±12.7(76) | 0.0±0.0(4) | 22.5±12.2(26) | 41.5±16.9(12) |
| 87-96 | 12-Mo RS |  |  |  |  |  |
|  | All | 86.0±2.7(195) | 87.2±1.3(793) | 94.8±2.1(131) | 78.4±2.6(289) | 96.5±0.8(697) |
|  | 0-44 | 86.7±5.6(37) | 92.3±2.9(89) | 98.4±1.6(62) | 90.2±5.5(30) | 96.7±1.5(145) |
|  | 45-59 | 79.9±7.0(44) | 92.3±1.9(215) | 94.4±4.0(34) | 90.5±3.4(80) | 97.5±1.1(251) |
|  | 60-74 | 79.3±6.9(69) | 86.9±2.1(303) | 94.1±5.3(26) | 80.8±4.0(109) | 96.4±1.5(245) |
|  | 75+ | 52.3±7.9(45) | 79.4±3.5(186)* | 68.9±16.2(9) | 54.4±6.3(70) | 90.3±4.9(56)** |
|  | 60-Mo RS |  |  |  |  |  |
|  | All | 72.0±3.8(195) | 74.3±2.0(793) | 81.7±3.9(131) | 67.2±3.2(289) | 94.0±1.4(697)*** |
|  | 0-44 | 81.5±6.5(37) | 88.9±3.5(89) | 95.5±2.8(62) | 80.4±7.3(30) | 94.5±2.0(145) |
|  | 45-59 | 98.4±2.3(44) | 83.7±2.8(215) | 76.2±7.8(34) | 80.4±4.9(80) | 96.5±1.7(251) |
|  | 60-74 | 82.9±4.8(69) | 69.1±3.2(303) | 65.4±11(26) | 64.6±5.3(109) | 89.9±3.0(245) |
|  | 75+ | 76.4±6.9(45) | 62.1±5.8(186) | 35.2±22.0(9) | 47.5±8.1(70)* | 90.3±4.9(56) |
|  | 120-Mo RS |  |  |  |  |  |
|  | All | 62.9±4.6(195) | 61.2±2.4(793) | 74.2±4.7(131) | 61.4±3.8(289) | 90.0±2.0(697)*** |
|  | 0-44 | 79.9±7.0(37) | 87.1±4.0(89) | 91.7±3.9(62) | 80.4±7.3(30) | 92.1±2.6(145) |
|  | 45-59 | 86.5±5.7(44) | 69.7±3.7(215) | 94.2±8.8(34) | 74.0±5.8(80) | 94.0±2.5(251) |
|  | 60-74 | 69.7±6.5(69) | 51.8±3.9(303) | 35.5±11.5(26)* | 63.8±6.5(109) | 84.3±4.1(245) |
|  | 75+ | 49.7±10.0(45) | 46.3±7.4(186) | 35.2±22.0(9) | 23.4±8.4(70) | 81.9±15.1(56)* |
| 97-06 | 12-Mo RS |  |  |  |  |  |
|  | All | 84.6±1.7(516) | 92.3±0.8(1417) | 94.9±1.3(323) | 80.9±1.7(605) | 98.1±0.4(1762)*** |
|  | 0-44 | 95.4±2.6(65) | 98.0±1.3(134) | 98.6±1.1(133) | 87.8±4.1(65) | 98.7±0.7(278) |
|  | 45-59 | 90.5±2.4(160) | 96.6±0.9(467) | 95.5±2.0(123) | 87.6±2.3(208) | 98.9±0.4(888) |
|  | 60-74 | 87.2±2.8(161) | 92.4±1.3(529) | 91.0±4.6(48) | 78.3±3.0(211) | 97.6±0.9(476) |
|  | 75+ | 68.4±4.5(130) | 82.6±2.6(287)* | 72.9±11.4(19) | 69.9±4.5(121) | 92.2±3.3(120)*** |
|  | 60-Mo RS |  |  |  |  |  |
|  | All | 76.4±2.3(516) | 83.4±1.3(1417)* | 86.2±2.2(323)* | 68.6±2.2(605) | 96.4±0.8(1762)*** |
|  | 0-44 | 85.2±4.6(65) | 91.9±2.5(134) | 96.0±1.8(133) | 76.0±5.4(65) | 96.9±1.1(278) |
|  | 45-59 | 83.6±3.2(160) | 97.3±1.8(467)* | 80.4±3.8(123) | 78.6±3.1(208) | 97.5±0.8(888)** |
|  | 60-74 | 75.8±4.1(161) | 83.1±2.1(529) | 95.1±7.5(48) | 65.0±3.8(211) | 96.5±1.7(476)*** |
|  | 75+ | 61.1±5.6(130) | 71.3±4.0(287) | 72.9±11.4(19) | 51.9±6.2(121) | 84.2±6.5(120)** |
|  | 120-Mo RS |  |  |  |  |  |
|  | All | 68.8±2.8(516) | 71.4±1.7(1417) | 77.9±2.8(323) | 64.5±2.6(605) | 96.3±0.8(1762)*** |
|  | 0-44 | 83.0±5.0(65) | 84.7±3.4(134) | 93.7±2.4(133) | 72.0±5.8(65) | 95.6±1.5(278) |
|  | 45-59 | 78.3±3.7(160) | 76.8±2.3(467) | 68.4±4.7(123) | 73.7±3.5(208) | 97.2±0.9(888)*** |
|  | 60-74 | 64.2±5.1(161) | 69.1±2.8(529) | 65.1±9.3(48) | 62.9±4.1(211) | 96.4±1.8(476)*** |
|  | 75+ | 49.3±8.4(130) | 55.9±6.0(287) | 36.0±19.1(19) | 41.8±8.2(121) | 84.2±6.5(120)*** |
| 07-16 | 12-Mo RS |  |  |  |  |  |
|  | All | 91.1±1.0(987) | 95.7±0.4(3285) | 97.7±0.4(1444)* | 80.3±1.4(932)*** | 98.8±0.2(3774)** |
|  | 0-44 | 94.8±2.0(134) | 99.7±0.4(276) | 99.5±0.3(674) | 90.4±3.5(73) | 98.7±0.5(508) |
|  | 45-59 | 93.9±1.4(337) | 97.8±0.5(1111) | 97.5±0.8(434) | 88.0±1.7(403) | 99.0±0.3(2180) |
|  | 60-74 | 91.4±1.7(336) | 95.5±0.7(1284) | 96.6±1.3(277) | 81.5±2.3(312) | 98.8±0.5(947) |
|  | 75+ | 80.8±3.2(180) | 90.4±1.5(614) | 84.4±5.0(59) | 84.4±5.0(144) | 93.1±2.9(139) |
|  | 60-Mo RS |  |  |  |  |  |
|  | All | 83.3±1.6(987) | 88.6±0.9(3285) | 90.5±1.1(1444)* | 69.9±1.8(932)*** | 97.6±0.4(3774)*** |
|  | 0-44 | 89.8±2.9(134) | 97.5±1.3(276) | 97.5±0.9(674) | 80.1±5.0(73) | 97.3±0.8(508) |
|  | 45-59 | 88±2.1(337) | 92.6±1.0(1111) | 86.2±2.0(434) | 80.4±2.2(403) | 97.9±0.5(2180)* |
|  | 60-74 | 82.9±2.7(336) | 85.8±1.4(1284) | 86.2±3.0(277) | 68.6±3.1(312)* | 97.8±1.1(947)*** |
|  | 75+ | 69.1±5.1(180) | 82.5±3.2(614)* | 66.2±11.2(59) | 35.9±5.5(144) | 85.7±4.2(139) |
|  | 120-Mo RS |  |  |  |  |  |
|  | All | 81.9±2.2(987) | 79.1±1.7(3285) | 82.8±2.2(1444) | 65.8±2.4(932)*** | 97.4±0.7(3774)*** |
|  | 0-44 | 86.7±3.7(134) | 94.2±2.6(276) | 93.0±2.3(674) | 74.1±6.5(73) | 95.0±1.5(508) |
|  | 45-59 | 84.9±3.3(337) | 84.5±2.0(1111) | 78.5±3.4(434) | 77.1±2.9(403) | 97.9±0.5(2180)*** |
|  | 60-74 | 81.6±3.0(336) | 73.6±2.6(1284) | 73.0±5.9(277) | 62.8±4.6(312)*** | 96.3±1.4(947)*** |
|  | 75+ | 63.4±8.4(180) | 69.5±7.5(614) | 52.4±10.0(59) | 29.6±8.1(144)*** | 83.4±10.0(139)* |

Abbreviations: Mo, month; RS, relative survival; SEM, standard error of the mean.

*P < 0.01, **P < 0.001, and ***P < 0.0001 for comparisons with the Stomach group.
